# Supplementary material for: Robustly learning the Hamiltonian dynamics of a superconducting quantum processor
Source: Nat Commun. 2024 Nov 6;15:9595. doi: 10.1038/s41467-024-52629-3 (PMC11542007; doi:10.1038/s41467-024-52629-3)
Supplement: Supplementary file 1 — Supplementary Information [file 41467_2024_52629_MOESM1_ESM.pdf]

Supplemental material for
"Robustly learning the Hamiltonian dynamics of a superconducting quantum processor"

Dominik Hangleiter, Ingo Roth, Jonáš Fuksa, Jens Eisert, and Pedram Roushan

CONTENTS

I. Overview 1

II. Frequency extraction via rotational invariance 2
A. ESPRIT 2
B. Tensor ESPRIT 3

III. Reconstruction of eigenvectors 4
A. Linear inversion with post-projection 5
B. Non-convex manifold optimization 5

IV. Addressing SPAM errors 8
A. Removing and characterizing SPOM errors 8
B. The measurement error 8

V. The complete identification algorithm 9
A. Estimating the time complexity 9
B. Increasing the predictive power 10

VI. Numerical benchmarks 10
A. Data models and Hamiltonian ensembles 10
B. Frequency extraction 11
C. Eigenspace reconstruction 12
D. Compressed sensing capabilities 13

VII. Estimating experimental errors 14
A. Systematic error: Final ramp effect estimation 14
B. Statistical error: Bootstrapping 17

VIII. Reconstructing diagonal orthogonal final maps 17

References 18

I. OVERVIEW

In this supplemental material, we elaborate on the details of the identification algorithm, provide numerical benchmarks for it, and discuss in more detail how we estimate the statistical and systematic errors on the Hamiltonian identified in the experiment.

Recall that our ideal data model—in the absence of *state-preparation and measurement* (SPAM) errors is given by (cf. Eq. (3) of the main text)

$$y[l] = \frac{1}{2}\exp(-\mathrm{i}t_l h), \tag{1}$$

where t_l, $l = 0,\dots,L$ are the time stamps, and h is the $N\times N$ coefficient matrix of the non-interacting Hamiltonian given in Eq. (2). Our identification technique follows the standard approach of Fourier analysis of time series signals but at the same time aims to maximally exploit all structure present in the signal. Our starting point is the eigendecomposition $h = \sum_{k=1}^{N} \lambda_k \left|v_k\right\rangle\left\langle v_k\right|$ of the coefficient matrix h, where λ_k are the eigenvalues and $\left|v_k\right\rangle$ the eigenvectors. Using the eigendecomposition we diagonalise the signal as

$$\begin{aligned} y[l] &= \frac{1}{2}\exp(-\mathrm{i}t_l h) \\ &= \frac{1}{2}\sum_{k=1}^{N} \mathrm{e}^{-\mathrm{i}t_l\lambda_k}\left|v_k\right\rangle\!\left\langle v_k\right|. \end{aligned} \tag{2}$$

From a signal processing perspective, the time series of each entry $y_{m,n}$ is therefore given by a complex linear superposition of a small number of sinusoids. But additional constraints relate the different time series. As discussed in the main text, our identification technique proceeds in two steps: In the *first step*, we estimate the frequency spectrum of the time series, i.e., the eigenvalues λ_k of h. Crucially, we can exploit that the signal is sparse in Fourier space and also that $y[l]$ are noisy samples from a one-parameter subgroup of the unitary group to dramatically denoise the signal and arrive at sub-Nyquist resolution. Given the frequency spectrum of the time series data, the estimation of the Fourier coefficients becomes a linear inverse problem. In standard Fourier data analysis the Fourier coefficients are therefore typically inferred via linear inversion in the *second step*. However, in the case of Hamiltonian recovery, the problem has a considerably richer structure that we can take advantage of. The N^3 Fourier coefficients are certain second order polynomials of the entries of a set of *orthogonal vectors* $\{\left|v_k\right\rangle\}_{k=1}^{N}$. Furthermore, if the interaction graph is not fully connected, the resulting sparsity pattern of the Hamiltonian matrix gives rise to linear constraints on the coefficients.

As we will see, exploiting the structure in the second step brings two benefits in terms of noise robustness: First, explicitly enforcing the orthogonality constraint, polynomial structure (low-rank) and the linear constraints from the interaction graph renders the reconstruction significantly more robust against different sources of errors such as incoherent measurement errors. Second, since the coefficient matrices for each frequency are unit rank projectors, we can significantly denoise the signal from systematic (state-preparation) errors on the projectors by restricting ourselves to inferring the projector's range first, i.e., $\{\left|v_k\right\rangle\}_{k=1}^{n}$. Only then do we infer its domain, that is, the state preparation map. This yields robustness to *state preparation or measurement* (SPOM) errors S or M in the data model (cf. Eq. (4) of the main text). Both ways of denoising the signal from coherent and incoherent noise are crucial for our reconstruction method to be practically applicable.

In the following, we detail algorithmic strategies for the different steps of our identification algorithm, relying on ad-

vanced state-of-the-art signal processing techniques that are capable of exploiting the entire structure of the problem, as well as developing new tools tailored to this specific structure. For the sake of clarity we first consider the ideal data model (1), and then discuss the effect and removal of SPAM errors in the algorithm.

We begin in Section II by introducing a novel super-resolving and denoising algorithm *tensorESPRIT* that is able to scalably resolve arbitrary degenerate frequency spectra of Hermitian matrices from samples of the corresponding one-parameter subgroup of the unitary group. Given the eigenfrequencies, in Section III we discuss and compare different ways of reconstructing the eigenvectors of h in the presence of constraints. In Section IV we then discuss the SPAM errors that affect the ideal data, and elaborate ways of partially removing those errors, giving rise to a robust recovery algorithm. In Section V we summarize the entire algorithm, before benchmarking its performance in Section VI. Finally, in Section VII we explain how we obtain the systematic and statistical error bars for the plots in the main text.

II. FREQUENCY EXTRACTION VIA ROTATIONAL INVARIANCE

The data of the form as elaborated upon in (2) consists of N^2 time series, each described by a linear combination of N sinusoids oscillating at the eigenfrequencies of the Hamiltonian, which we wish to recover. A simple approach to recover these frequencies would be to use Fourier analysis. This approach is limited by different imperfections since some of the Fourier coefficients can become small. Furthermore, it is far from obvious how to combine the spectra recovered from each time series, especially if the spectrum is degenerate or nearly degenerate. Additionally, Fourier approaches are typically limited in precision by Shannon's sampling theorem [1]. Since we need the spectrum very accurately in order for the eigenvector reconstruction to converge, we need a more sophisticated approach.

To arrive at such a method, we make two structural observations. Firstly (*sparsity*), we notice that the spectrum is sparse in Fourier space; there are exactly N frequencies to be recovered. This is a significantly more constraining structure than Shannon's band limitation alone. Secondly (*rotational-invariance*), the matrix time series $y_{m,n}[l]$ are noisy samples from the one-parameter subgroup of the unitary group $\mathrm{U}(N)$ generated by h and parameterised by time.

It will be useful to first think about the scalar ($N = 1$) case where powerful algorithms exist, before extending those approaches to the matrix case with $N > 1$. The problem of extracting sparse frequencies (or also decay parameters, i.e. complex poles) from discrete scalar time series has been well studied for centuries [2]. Modern stable algorithms for finding solutions to this problem have been devised in the context of direction-of-arrival estimation in array signaling [3, 4]. Here the goal is to find directions of arrival of a sparse set of electromagnetic signals from noisy snapshots detected by an array of antennas in the far field. Exploiting *sparsity* and *rotational* $\mathrm{U}(1)$ *invariance*, these methods utilize signal space estimation, denoising and low-rank Hankel structure to achieve precision beyond Shannon's theorem, which is a phenomenon called *super-resolution*. Theoretical understanding of super-resolution has only been developed recently [5, 6].

A. ESPRIT

A particularly elegant example, relying solely on linear algebra routines, is the ESPRIT algorithm proposed in Ref. [7] and analysed in Refs. [8, 9]. Let $t_l = l\Delta t$ be equally spaced with $l \in \{0, \dots, L\}$. ESPRIT then assumes time series data of the form

$$x[l] = \sum_{k=1}^{N} x_k \mathrm{e}^{-i\lambda_k l \Delta t} = \sum_{k=1}^{N} x_k z_k^l, \qquad (3)$$

where $z_k \coloneqq \mathrm{e}^{-i\lambda_k \Delta t}$. The first step of the ESPRIT algorithm is to form the *Hankel matrix*

$$\mathrm{Hk}_K(x) = \begin{pmatrix} x[0] & x[1] & \dots & x[L-K] \\ x[1] & x[2] & \dots & \\ \vdots & & \ddots & \\ x[K] & x[K+1] & \dots & x[L] \end{pmatrix}, \qquad (4)$$

of the scalar time series (3), defined for an integer $0 < K < L$. The Hankel matrix admits Vandermonde decomposition

$$\mathrm{Hk}_K(x) = \Psi^K(z)\,\mathrm{diag}(x)\left(\Psi^{L-K}(z)\right)^T, \qquad (5)$$

where the Vandermonde matrix $\Psi^J(.)$ for an integer J is given by

$$\Psi^J(z) \coloneqq \begin{pmatrix} 1 & 1 & \dots & 1 \\ z_1 & z_2 & \dots & z_N \\ z_1^2 & z_2^2 & \dots & z_N^2 \\ \vdots & \vdots & \ddots & \vdots \\ z_1^J & z_2^J & \dots & z_N^J \end{pmatrix} \in \mathbb{C}^{(J+1)\times N}. \qquad (6)$$

This decomposition makes the rotational invariance of the data apparent. To see this, notice that inserting $\mathrm{diag}(z)$ between the first and second matrix in (5) of the decomposition gives rise to a Hankel matrix of shifted data $x[l]$ for $l = 1, \dots, L+1$.

Denote by $\mathrm{Hk}_K^{\downarrow(\uparrow)}(x) \in \mathbb{C}^{K\times(L-K+1)}$ the matrices obtained from $\mathrm{Hk}(x)_K \in \mathbb{C}^{(K+1)\times(L-K+1)}$ by deleting the last (first) row. We can write

$$\begin{aligned} \mathrm{Hk}_K^{\downarrow}(x) &= \Psi^{L-1}(z)\,\mathrm{diag}(x)\left(\Psi^{L-K}(z)\right)^T, \\ \mathrm{Hk}_K^{\uparrow}(x) &= \Psi^{K-1}(z)\,\mathrm{diag}(z)\,\mathrm{diag}(x)\left(\Psi^{L-K}(z)\right) 1. \end{aligned} \qquad (7)$$

Note that $\mathrm{diag}(z), \mathrm{diag}(x) \in \mathbb{C}^{N\times N}$. It can be shown that the Vandermonde matrices have full row rank if the frequencies are non-degenerate and $N + 1 \le K \le L - N$. Hence, in this case, we have

$$\mathrm{rank}\left(\mathrm{Hk}_K^{\downarrow(\uparrow)}(x)\right) = \mathrm{rank}\left(\mathrm{Hk}_K(x)\right) = N\,. \qquad (8)$$

We can, thus, denoise the data by using the rank-N approximation of $\mathrm{Hk}_K(x)$,

$$\widehat{\mathrm{Hk}}_K(x) \coloneqq U_{[N]}\Sigma_{[N]}V_{[N]}^{\dagger}, \tag{9}$$

where $U\Sigma V^{\dagger}$ is the *singular value decomposition* (SVD) of $\mathrm{Hk}_K(x)$ and the subscript $[N]$ denotes restriction to the subspace of the N dominant singular values. From the denoised Hankel matrix we can obtain the denoised shifted matrices $\widehat{\mathrm{Hk}}_K^{\downarrow(\uparrow)}(x)$.

The decompositions (7) together with the rank considerations imply that

$$\Psi \coloneqq \left(\widehat{\mathrm{Hk}}_K^{\downarrow}(x)\right)^{+} \widehat{\mathrm{Hk}}_K^{\uparrow}(x), \tag{10}$$

where $(\cdot)^{+}$ denotes the pseudoinverse, has in absence of noise non-vanishing eigenvalues $\{z_1, \ldots, z_N\}$. The eigenvalues of Ψ therefore provide an estimate of the eigenfrequencies $\{\lambda_k\}_{k=1}^{N}$, if the time step Δt is chosen such that there exists a branch cut of the complex logarithm that uniquely identifies λ_k from z_k for each k.

In order to apply the ESPRIT algorithm to the data of the Hamiltonian identification problem, we compute a suitable scalar time series from the matrix-valued time series (1). Optimal performance of ESPRIT in resolving the spectrum is expected when the Fourier coefficients of the time-series are as large as possible and of the same order of magnitude [9]. This is particularly important for large N with unavoidably many adjacent frequencies. In the data model (1), we can achieve this by taking the matrix trace at each time

$$F[l] = \mathrm{Tr}\,[y[l]] = \frac{1}{2}\sum_{k=1}^{N} \mathrm{e}^{-i\lambda_k l\Delta t}. \tag{11}$$

In practice, imperfections in the state-preparation and measurements (due to finite speed of control) will alter the data model (1). We describe a pre-processing procedure to still get a signal of approximately the form (11) in the presence of SPOM in Section IV A.

The time complexity of ESPRIT is dominated by calculating the SVD which for an $m \times n$ matrix requires $\mathcal{O}(\min\{m^2 n, mn^2\})$ flops, e.g., using Householder reflections [10]. For the Hankel dimension $K \in \mathcal{O}(N)$ ESPRIT, thus, takes time $\mathcal{O}(N^2 L)$, with L the length of the time series and N the number of sinusoids. Memory cost stays of the order of the size of the input $\mathcal{O}(N^2 L)$.

B. Tensor ESPRIT

ESPRIT can, in principle, recover frequencies that are considerably closer together than the Shannon-Nyquist rate. For increasing system sizes N the spectrum becomes unavoidably closer and closer to being degenerate (if one does not increase the energy scale extensively with the system size). For this reason, for sufficiently large N the approach of reducing the data to a single scalar time-series and running ESPRIT will inevitably fail. We here develop a novel algorithm that directly works on the matrix time series and makes use of the entire $\mathrm{U}(N)$-rotational invariance of the data. To this end, we utilize tensor-network methods, carefully exploiting the properties of the data series over different contractions in the tensor network. The new *tensorESPRIT* algorithm is capable of resolving even degenerate Hamiltonian spectra and, thus, enables finding the complete eigenfrequency spectrum even of large-size instances.

Algorithm 1 $\mathrm{ESPRIT}(F, K)$ (frequency extraction)

Input: $F \in \mathbb{C}^{(L+1)}$, $K \le L$.
1: Set $H = \mathrm{Hk}_K(F)$.
2: Calculate the SVD of $H = U\Sigma V^{\dagger}$.
3: Set $P_{\text{signal}} = U_{[N]}(U_{[N]})^{\dagger}$.
4: Calculate $\Psi = (P_{\text{signal}} H^{\uparrow})^{+} H^{\downarrow}$.
5: Calculate $z = \mathrm{eigenvalues}(\Psi)$.
Output: z.

With $t_l = l\Delta t$ equally spaced with $l \in \{0, \ldots, L\}$, the time series (2) takes the form

$$y[l] = \frac{1}{2}\sum_{p=1}^{N} z_p^{l}\, |v_p\rangle\langle v_p|, \tag{12}$$

where again $z_p \coloneqq e^{-i\Delta t\lambda_p}$. Let $Q = \sum_p |v_p\rangle\langle p|$ be an orthogonal matrix with columns $|v_p\rangle$. We form the Hankel tensor, which we define to be

$$\mathrm{Hk}_K(y)_{m,k,l,n} \coloneqq 2y_{m,n}[k+l] = \sum_{p=1}^{N} z_p^{k+l} Q_{m,p} Q_{n,p} \tag{13}$$

with $k \in \{0, \ldots, K\}$ and $l \in \{0, \ldots, L-K\}$. Due to rotational invariance of the data, $\mathrm{Hk}_K(y)$ admits a tensor version of the Vandermonde decomposition. In tensor network notation[1] we find

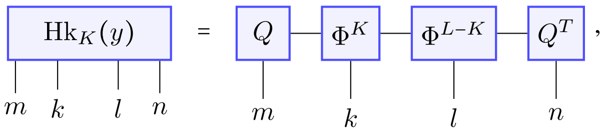

(14)

where Φ^J for an integer J is the Vandermonde tensor with components

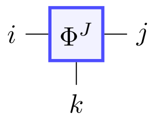

$\coloneqq \delta_{i,j} z_i^{k}, \quad k \in \{0, \ldots, J\}.$ (15)

[1] In this notation, a degree k tensor is represented by a box with k legs attached to it, representing the indices. If two boxes are connected by a leg, the corresponding index is contracted over. See Ref. [11] for a thorough introduction to this notation.

We will obtain an unfolding $\mathcal{H}_K(y) \in \mathbb{C}^{(K+1)N\times(L-K+1)N}$ of $\mathrm{Hk}_K(y)$ by grouping together the indices m,k and l,n, respectively, such that the $N\times N$ block structure of $\mathcal{H}_K(y)$ is

$$\mathcal{H}_K(y) = \begin{pmatrix} y[0] & y[1] & \dots & y[L-K] \\ y[1] & y[2] & \dots & \\ \vdots & & \ddots & \\ y[K] & y[K+1] & \dots & y[L] \end{pmatrix}. \tag{16}$$

Decomposition (14) implies that $\mathcal{H}_K(y)$ can be written as the product of an $NK\times N$ matrix, the correspondingly unfolded $Q^T\Phi^K$, and an $N\times N(M-L)$ matrix, the unfolded $\Phi^{L-K}Q$. Hence the rank of $\mathcal{H}_K(y)$ is upper bounded by N, which allows us to denoise the data by applying a rank-N approximation to $\mathcal{H}_K(y)$, defined in (9).

Similarly to ESPRIT, we define shifted tensors

$$\begin{aligned} \mathrm{Hk}^{\downarrow}_K(y)_{m,k,l,n} &\coloneqq \mathrm{Hk}_K(y)_{m,k,l,n}, \\ \mathrm{Hk}^{\uparrow}_K(y)_{m,k,l,n} &\coloneqq \mathrm{Hk}_K(y)_{m,k+1,l,n} \end{aligned} \tag{17}$$

with the restriction $k \in 0,\dots,K-1$ in both cases. These tensors admit Vandermonde decompositions

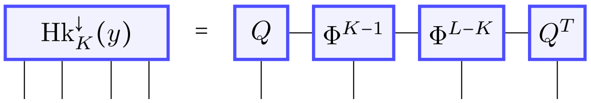

(18)

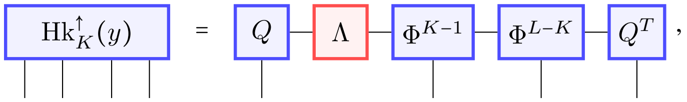

(19)

where $\Lambda = \mathrm{diag}(z)$. Now we make the crucial observation that in the noiseless case for any fixed k,l the matrix

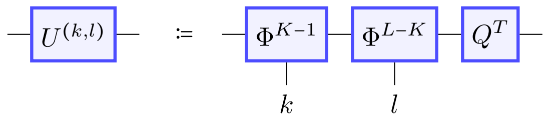

(20)

is unitary. Furthermore $U^{(k,l)} = U^{(k',l')}$ whenever $k+l = k'+l'$. Choosing k,l,k',l' such that $k+l = k'+l'$, we compute the matrix

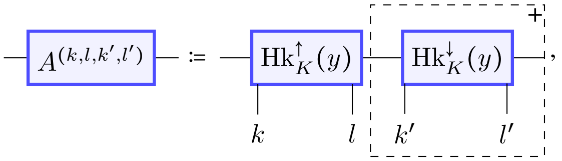

(21)

where the box with the plus denotes that we fix all the indices inside the box and then take the psuedoinverse of the matrix indexed by legs going through the box. From the observation (20) we conclude that (in the absence of noise) $A^{(k,l,k',l')} = Q^T\Lambda Q$ has spectrum $\{z_i\}_{i=1}^n$.

It may seem that we could have used the much cheaper Hermitian conjugate instead of the pseudoinverse in (21). But in the presence of noise, $U^{(k,l)}$ can deviate from being unitary, and hence using the pseudoinverse leads to a more accurate recovery. In fact, tensorESPRIT with the pseudoinverse can be applied more generally even if Q and Q^T are replaced by two (potentially different) invertible matrices, such as in the case of our SPAM model (50).

Algorithm 2 tensorESPRIT$(y,K,\mathcal{S})$ (frequency extraction)

Input: $y \in \mathbb{C}^{N\times N\times(L+1)}$, $K \le L$, $\mathcal{S}$.

Set $\mathcal{H}_K$ to be the unfolding (16) of $\mathrm{Hk}_K(y)$.

Set $\widehat{\mathcal{H}} = U_{[N]}\Sigma_{[N]}V^{\dagger}_{[N]}$ as the rank-N approximation of $\mathcal{H}_K$.

From $\widehat{\mathcal{H}}$ as Hankel tensor, calculate $A^{(k,l,k',l')}$ of (21) for all $(k,l,k',l') \in \mathcal{S}$.

Set $\hat{A} = |\mathcal{S}|^{-1}\sum_{\mathcal{S}} A^{(k,l,k',l')}$.

Calculate $z = \mathrm{eigenvalues}(\hat{A})$.

Output: z.

Combining the spectra of matrices $A^{(k,l,k',l')}$ for different choices of k,k',l,l' improves the noise robustness of the estimate. To this end, we define a set $\mathcal{S}$ of tuples (k,l,k',l') with $k+l = k'+l'$, and compute

$$\hat{A} = \frac{1}{|\mathcal{S}|}\sum_{(k,l,k',l')\in\mathcal{S}} A^{(k,l,k',l')}. \tag{22}$$

The spectrum of $\hat{A}$ is the arithmetic mean of the spectra of $A^{(k,l,k',l')}$ for $(k,l,k',l') \in \mathcal{S}$ and yields a robust estimate of $\{z_i\}_{i=1}^n$. We find empirically that

$$\mathcal{S} = \{(k,l,k',l') :\ 0 \le k = k' < K,\ 0 \le l = l' \le L-K\} \tag{23}$$

gives good performance and we use this definition throughout this manuscript.

As noted above, tensorESPRIT is robust against non-singular S and M in the SPAM data model (50). Poor conditioning of these matrices however still reduces its accuracy. Hence, we can apply tensorESPRIT to the data $y[l]$ without the pre-processing (Section IV A) required for ESPRIT. The tensorESPRIT algorithm is summarized as Algorithm 2.

The time complexity of tensorESPRIT is $\mathcal{O}((LN^2+|\mathcal{S}|)\times N^3)$ flops, while memory complexity is $\mathcal{O}(LN^2)$—of the order of the size of the input, using implicit definition of the Hankel tensor. The first term in the time complexity comes from the SVD of the unfolded Hankel tensor, which with the choice $K \in \mathcal{O}(N)$ is an $\mathcal{O}(N^2)\times\mathcal{O}(LN)$ matrix, where we expect $L \gg N$. Using randomized truncated SVD methods [12], this term can be further improved to $\mathcal{O}(LN^3\log(N))$. The second term comes from the computation of the matrix $\hat{A}$ from $\widehat{\mathcal{H}}$. The maximal size of $\mathcal{S}$ is $\mathcal{O}(L^3)$, but the set $\mathcal{S}$ used in this work has size in $\mathcal{O}(L^2)$.

III. RECONSTRUCTION OF EIGENVECTORS

After extracting the eigenfrequencies $\{\lambda_k\}$, in the second step of the Hamiltonian identification, we infer the eigenvec-

tors of h. Let Ω be the support set of the matrix h of the Hamiltonian model as determined by the interaction graph of h. This set comprises of index pairs (i,j) corresponding to a hopping term between sites i and j. For example, for the non-interacting Bose-Hubbard model (Eq. (1) of the main text) with nearest neighbour interaction, the support is given by the diagonal and first-order off-diagonal terms. For a support set Ω, $\bar{\Omega}$ denotes the complement of its support. For a matrix X, X_Ω denotes the sub-matrix restricted to the entries in Ω and $\|X\|_F = \sum_{i,j}|X_{i,j}|^2$ the Frobenius norm.

The eigenvector recovery task can be formulated as the least-squares optimization problem

$$\begin{aligned} \underset{\{|v_i\rangle\}}{\text{minimise}} \quad & \sum_l \left\| y[l] - \sum_k \mathrm{e}^{-\mathrm{i}\lambda_k t_l} |v_k\rangle\langle v_k| \right\|_F^2 , \\ \text{subject to} \quad & \langle v_i|v_j\rangle = \delta_{i,j}, \quad \left(\sum_k \lambda_k |v_k\rangle\langle v_k|\right)_{\bar{\Omega}} = 0. \end{aligned} \tag{24}$$

The objective function is a quartic polynomial. In addition, we encounter a non-convex constraint enforcing the orthogonality and quadratic support enforcing the sparsity.

To simplify the expressions it is helpful to introduce the following notation. Let $\mathrm{vec}: \mathbb{C}^{d\times d'} \to \mathbb{C}^{d\cdot d'}$ denote row-wise vectorization, which acts as $|i\rangle\langle j| \mapsto |i\rangle\,|j\rangle$ on the orthogonal basis. Using row-wise vectorization, we rewrite the data $y[l] \in \mathbb{C}^{N\times N}$ with $l \in [L]$ as a single $(L+1)\times N^2$ matrix

$$Y = \begin{pmatrix} \mathrm{vec}(y[0])^T \\ \mathrm{vec}(y[1])^T \\ \vdots \\ \mathrm{vec}(y[L])^T \end{pmatrix}. \tag{25}$$

Let $Q = \sum_k |v_k\rangle\langle k|$ be an orthogonal matrix with columns $|v_k\rangle$. We define the map $\Pi : \mathrm{O}(N) \to \mathbb{R}^{N\times N^2}$ on the orthogonal group $\mathrm{O}(N)$ as

$$\Pi(Q) := \sum_k |k\rangle\,\langle v_k|\,\langle v_k|\,. \tag{26}$$

This definition is equivalent with

$$\langle k|\,\Pi(Q)\,|l\rangle\,|m\rangle = \Pi(Q)_{k,(l,m)} = Q_{l,k}Q_{m,k}\,. \tag{27}$$

Furthermore, let A^λ be the matrix with the time series $l \mapsto \mathrm{e}^{-\mathrm{i}\lambda_k t_l}$ as its k-th column. For equidistant times, A^λ is the previously encountered Vandermonde matrix. Lastly, we define $H^\lambda : \mathbb{R}^{N\times N^2} \to \mathbb{R}^{N\times N}$ that given $\Pi(Q)$ returns the Hamiltonian matrix associated to Q with eigenvalues λ. Explicitly, $H^\lambda(\Pi) = \mathrm{vec}^{-1}(\sum_i \langle i|\,\mathrm{diag}(\lambda)\Pi)$.

In this notation the optimization problem (24) can be recast more compactly as

$$\begin{aligned} \underset{Q\in\mathrm{O}(N)}{\text{minimise}} \quad & \left\|Y - A^\lambda \Pi(Q)\right\|_F^2 , \\ \text{subject to} \quad & (H^\lambda(\Pi(Q)))_{\bar{\Omega}} = 0\,. \end{aligned} \tag{28}$$

In the following, we detail two distinct strategies for solving the optimization problem (28). First, we solve the problem for $\Pi(Q)$ using linear inversion in Section III A. We can further enforce the projector structure of the rows of $\Pi(Q)$ by post-projection, but not their orthogonality. Second, we make use of gradient-descent methods constrained to the non-convex manifold of orthogonal matrices in Section III B. We approximately incorporate the locality constraint by regularization.

A. Linear inversion with post-projection

Of course, knowing the eigenspace projectors $\Pi(Q)$ and λ is already sufficient to calculate the corresponding Hamiltonian matrix. The simplest approach to the optimization problem is thereby to neglect the exact dependency of Π on Q, the non-convex constraint and the support constraint. This yields the significantly simpler optimization problem

$$\underset{\Pi\in\mathbb{C}^{N\times N^2}}{\text{minimise}} \quad \left\|Y - A^\lambda \Pi\right\|_F^2 \tag{29}$$

where we slightly overloaded the symbol Π. The linear inverse problem (29) can be solved in closed form as

$$\Pi = (A^\lambda)^+ Y\,. \tag{30}$$

Note that in principle it is also straight-forward to solve for the linear support constraint when setting-up the optimization problem.

This matrix will in general not retain the projector structure of the signal, however. Indeed, recall that $\Pi = \Pi(Q)$ with $Q = \sum_l |l\rangle\,\langle v_l| \in \mathrm{O}(N)$, so the l-th row of Π is the vectorization of the projector onto the eigenspaces spanned by $|v_l\rangle$. In order to enforce this structure, we make use of a post-projection step: Given a matrix P, we can project it onto the manifold of real unit-rank projectors in order to enforce this constraint. To achieve this, we project them to Hermitian matrices $P' = (P + P^\dagger)/2$, perform an eigenvalue decomposition $P' = U\Lambda U^\dagger$, select the eigenvector U_1 of the absolutely largest eigenvalue and calculate $P'' = \mathrm{Re}\{U_1 U_1^\dagger\}$. We summarize the corresponding algorithm in Algorithm 3. Notice that the resulting projectors will in general not be mutually orthogonal.

The run-time of the algorithm scales as $\mathcal{O}(LN^2 + N^3)$, where the first term comes from the pseudo-inversion and the second term from performing N unit-rank projections of $N \times N$ matrices. Both steps can be implemented, e.g., using an SVD.

B. Non-convex manifold optimization

Taking the structure of the reconstruction problem for the eigenvectors (24) seriously requires us to account for non-convex orthonormality constraints. In the following, we detail how the optimization problem with orthonormality constraints can be solved using geometrical optimization techniques over the manifold structure that the orthogonal group exhibits as a Lie group. See, e.g., Refs. [13, 14] for a general introduction and a further reference, and Refs. [15–18] for manifold

Algorithm 3 $\mathrm{linInvPP}(y, A^\lambda, \lambda)$

Input: Data y, map A^λ.
Calculate $\Pi = (A^\lambda)^+ Y$.
for $k \in [N]$ **do**
  Set $P_k = \Pi[k,:]$ and reshape to $N \times N$ matrix.
  Project $P_k \leftarrow (P_k + P_k^\dagger)/2$
  Calculate u_k the eigenvector to the largest eigenvalue of P_k.
  Set $P_k = U_k U_k^\dagger$.
  Vectorize P_k and set $\Pi[k,:] = P_k$.
end for
Output: Projector matrix Π.

optimization in the context of quantum information and technologies.

To this end, we first neglect the sparsity constraint in (28), and consider the optimization problem

$$\underset{Q \in \mathrm{O}(N)}{\text{minimise}} \quad f_{y,A^\lambda}(Q) = \frac{1}{2} \left\| Y - A^\lambda \Pi(Q) \right\|_F^2 . \tag{31}$$

Many standard first-order and second-order optimization algorithm, such as gradient descent methods or Newton's method, readily generalize to matrix manifolds by using the differential structure and Riemannian geometry provided by a suitable embedding [14]. In the following we regard $\mathrm{O}(N) \subset \mathbb{R}^{N\times N}$ as a submanifold of the Euclidean space defined by its standard embedding as a matrix group. We now formulate a conjugate gradient algorithm for optimizing f over $\mathrm{O}(N)$ proposed in Refs. [13, 19]. The conjugate gradient algorithm iterates the following basic steps: (i) At a point Q_k on the manifold, determine a search direction V_k from the current gradient of the objective function f and the conjugacy conditions to the previous search directions with respect to the Hessian of the objective function. (ii) Perform a line search to determine the next point Q_{k+1} as the minimum of f along a geodesic through Q_k in direction V_k.

Search direction and the gradient are elements of the tangent space of the manifold $\mathrm{O}(N)$. The tangent space of $\mathrm{O}(N)$ is given by $T_Q\,\mathrm{O}(N) = \{V \in \mathbb{R}^{N\times N} \mid V^T Q + Q^T V = 0\}$ and can be equipped with the Riemannian metric

$$\langle V, W\rangle_Q = \frac{1}{2} \operatorname{Tr}[V W^T], \tag{32}$$

$Q \in \mathrm{O}(N)$ and $V, W \in T_Q\,\mathrm{O}(N)$, which is induced by the Euclidean metric on the ambient space. The tangent space at the group identity $\mathrm{Id} \in \mathrm{O}(N)$ is given by skew-symmetric matrices and identified with the Lie algebra $o(N)$. For $V \in o(N) = T_{\mathrm{Id}}\,\mathrm{O}(N)$, we have that $\tilde{V} = VQ \in T_Q\,\mathrm{O}(N)$. The orthogonal projection onto $T_Q\,\mathrm{O}(N)$ with respect to the Euclidean metric is $\mathcal{P}_{T_Q\,\mathrm{O}(N)} : \mathbb{R}^{n\times n} \to T_Q\,\mathrm{O}(N)$,

$$Z \mapsto \frac{1}{2}(Z - Q Z^T Q). \tag{33}$$

Given a search direction $V \in T_Q\,\mathrm{O}(N)$, a natural way to move forward on the manifold is along the geodesic of the Levi-Cevita connection defined by $\langle\cdot,\cdot\rangle_Q$. For $\mathrm{O}(N)$ a closed form of a geodesic $\gamma_{Q,\tilde{V}}$ in direction $\tilde{V} = VQ \in T_Q\,\mathrm{O}(N)$ through point $Q \in \mathrm{O}(N)$ is given by the matrix exponential, that here coincides with the exponential map from Lie theory,

$$t \mapsto \gamma_{Q,\tilde{V}}(t) = \exp(Vt) Q . \tag{34}$$

Note that here $V \in o(N)$ instead of $\tilde{V}$ appears in the exponent. More generally, the notion of a retraction generalizes the idea of moving along the manifold in a search direction while still ensuring convergence of descent algorithms [14]. Employing other retractions such as the Cayley transformation or projection using the QR-decomposition avoids the numerically costly matrix exponential and can reduce the computational complexity of the optimization algorithm. We will not pursue these alternatives here.

To formulate the conjugacy condition between tangent vectors at different points of the manifold, we require the parallel transport of tangent vectors along geodesics. The vector $\tilde{W} = WQ \in T_Q\,\mathrm{O}(N)$ parallel transported along the geodesic $\gamma_{Q,\tilde{V}}$, (34), to $T_{\gamma_{Q,\tilde{V}}(t)}\,\mathrm{O}(N)$ is given by

$$\Gamma^t_{\gamma_{Q,\tilde{V}}}(\tilde{W}) = \mathrm{e}^{\frac{1}{2}Vt} W \mathrm{e}^{\frac{1}{2}Vt} Q . \tag{35}$$

For $\tilde{W} = \tilde{V}$ the direction of $\gamma_{Q,\tilde{V}}$ at $Q = \gamma_{\tilde{V}}(0)$ this expression reads

$$\Gamma^t_{\gamma_{Q,\tilde{V}}}(\tilde{V}) = \tilde{V} Q^T \gamma_{Q,\tilde{V}}(t) . \tag{36}$$

Ignoring the structure of the manifold and considering the standard embedding of $\mathrm{O}(N) \subset \mathbb{R}^{n\times n}$, we can calculate the gradient with respect to the Euclidean metric of the ambient space. This Euclidean gradient can be subsequently projected onto the tangent space of the manifold $\mathrm{O}(N)$ to get a search direction of a gradient descent algorithm. The Euclidean gradient for our optimization problem is calculated as follows. We define

$$g_{A^\lambda}(X) \coloneqq \frac{1}{2} \left\| Y - A^\lambda X \right\|_F^2 \tag{37}$$

and have $f_{y,A^\lambda} = g_{y,A^\lambda} \circ \Pi$. Then, by the chain rule, it holds that

$$\begin{aligned} (\nabla_E f_{y,A^\lambda}(Q))_{i,j} &= \left.\frac{\partial f_{y,A^\lambda}}{\partial Q_{i,j}}\right|_Q \\ &= \sum_{k,l,m} \left.\frac{\partial g_{A^\lambda}}{\partial X_{k,(l,m)}}\right|_{\Pi(Q)} \left.\frac{\partial \Pi_{k,(l,m)}}{\partial Q_{i,j}}\right|_Q . \end{aligned} \tag{38}$$

The outer derivative of the linear least-square problem is given by

$$\left.\frac{\partial g}{\partial X_{i,j}}\right|_X = -\operatorname{Re}\{(A^\lambda)^\dagger (Y - A^\lambda \cdot X)\}. \tag{39}$$

The inner-derivative can be read-off from (27) to be

$$\left.\frac{\partial \Pi_{k,(l,m)}}{\partial Q_{i,j}}\right|_Q = \delta_{i,l}\delta_{j,k} Q_{m,k} + \delta_{i,m}\delta_{j,k} Q_{l,k} . \tag{40}$$

Note that carefully considering the order of the contractions of (38) and the sparsity pattern of the quantities, allows one to evaluate the gradient without performing routines in the full high-dimensional tensor spaces. At point $Q \in \mathrm{O}(N)$, we infer the Riemannian gradient via the tangent space projection as

$$\begin{aligned} \nabla f(Q) &= P_{T_Q \mathrm{O}(N)}[\nabla_E f(Q)] \\ &= \frac{1}{2}[\nabla_E f(Q) - Q(\nabla_E f(Q))^T Q] \,. \end{aligned} \tag{41}$$

Given the previous search direction H_{k-1} at point Q_{k-1}, the step size t_{k-1}, and the gradient G_k at point Q_k, the new search direction is calculated as

$$H_k = -G_k + \gamma_k \hat{H}_{k-1} \,, \tag{42}$$

with $\gamma_k \in \mathbb{R}$ and $\hat{H}_{k-1} = \Gamma^{t_k}_{\gamma_{Q_{k-1},H_{k-1}}} H_{k-1}$, the previous search direction H_{k-1} is parallel transported from Q_{k-1} to Q_k. Exact conjugacy requires

$$\gamma_k = \frac{\mathrm{Hess}\, f_{y,A^\lambda}(G_k, \hat{H}_{k-1})}{\mathrm{Hess}\, f_{y,A^\lambda}(\hat{H}_{k-1}, \hat{H}_{k-1})} \tag{43}$$

and can be approximated using the Polak-Ribière formula that arises from the finite difference approximation to the Hessian

$$\gamma_k = \frac{\langle G_k - \hat{G}_{k-1}, G_k \rangle_{Q_k}}{\langle G_{k-1}, G_{k-1} \rangle_{Q_{k-1}}} \,. \tag{44}$$

It is convenient to instead of working with different tangent spaces $T_{Q_k}\mathrm{O}(N)$, to express the search directions and gradients directly in terms of the translated in $o(N)$ arising from right multiplication with Q_k^T. Let $g_k = G_k Q_k^T$ and $h_k = H_k Q_k^T$. Then a quick calculation shows that the update of the search direction can be expressed as

$$h_k = -g_k + \gamma_k h_{k-1} \,. \tag{45}$$

Following the proposal of Ref. [19], the quantity γ_k can be further approximated by

$$\gamma_k = \frac{\langle g_k - g_{k-1}, g_k \rangle_{\mathrm{Id}}}{\langle g_{k-1}, g_{k-1} \rangle_{\mathrm{Id}}} \,. \tag{46}$$

Finally, we update

$$Q_{k+1} = \exp(t_k h_k) Q_k, \tag{47}$$

with step size t_k determined by a line search algorithm introduced in Ref. [19] that approximates the minimum of the objective function along the direction of h_k with a low-order polynomial. Thereby, we find the optimal step size t_k with only few cost function evaluations. We summarize the conjugate gradient algorithm as Algorithm 4.

Algorithm 4 $\mathrm{conjGrad}(f, Q_0, \epsilon)$

Input: Objective function f, initial point $Q_0 \in \mathrm{O}(N)$, tolerance ϵ.

Set $k = 0$
repeat
  Calculate the Euclidean gradient $G_k^E = \nabla_E f(Q_k)$ (here using (38), (39), and (40)).
  Calculate the translated Riemannian gradient $g_k = G_k^E Q_k^T - (G_k^E)^T Q_k \in o(N)$.
  Calculate gradient norm $n_k = \langle g_k, g_k \rangle_{\mathrm{Id}}$
  if $k = 0$ **then**
    Set $h_k = -g_k$
  else
    Set $\gamma_k = (n_k - \langle g_k, g_{k-1} \rangle_{\mathrm{Id}})/n_{k-1}$.
    Determine search direction as $h_k = -g_k + \gamma_k h_{k-1}$
  end if
  Perform line-search to determine t_k as $\arg\min$ of $t \mapsto f(\exp(h_k t) Q_k)$.
  Set $Q_{k+1} = \exp(h_k t_k) Q_k$.
until $n_k < \epsilon$ at $k = \hat{k}$

Output: objective point $Q_{\hat{k}}$ and objective value $f(Q_{\hat{k}})$.

Regularization. Above, we have neglected the sparsity constraint. This has resulted in an unconstrained optimization problem over the non-convex manifold $\mathrm{O}(N)$. A straightforward way to include the model constraints on the support of the Hamiltonian term is via an additional regularization term in the objective function. Specifically, in the regularization we replace the optimization problem (28) by the problem

$$\underset{Q \in \mathrm{O}(N)}{\mathrm{minimise}} \quad f_{y,A^\lambda,\mu}(Q) := f_{y,A^\lambda}(Q) + \mu\, r_\Omega(Q) \tag{48}$$

with the regularizer

$$r_\Omega := \|(H^\lambda(\Pi(Q)))_{\bar{\Omega}}\|_F^2 \tag{49}$$

and $\mu > 0$. The rationale behind the choice of the Frobenius norm as opposed to, say, the ℓ_1-norm of the weight of h on the complement of the support set—which might be the natural choice, see Ref. [17]—is the following: While the ℓ_1-norm is the natural regularizer promoting sparsity of a matrix, it also leads to a badly conditioned optimization problem since the gradient is non-continuous. This introduces steep edges in the optimization landscape. Conversely, the Frobenius norm constitutes a smooth regularizer which fares much better in the gradient descent algorithm. Moreover, since we are only interested in minimizing the total weight on the complement of the support, the corresponding regularizer effectively acts as a—slightly reweighted—ℓ_1 norm.

We observe that the conditioning of the optimization problem becomes worse when the data deviates from the constraint. This means that making a suitable choice of μ is a challenging problem. If we choose μ too large, the optimization problem becomes badly conditioned and the algorithm does not converge. If we choose it too small, the constraint is not enforced. We therefore proceed by running the optimization algorithm for increasing, exponentially spaced values of μ until it does not converge. We then perform binary search over μ to find the largest value of μ such that the algorithm converges.

IV. ADDRESSING SPAM ERRORS

As discussed in detail in the main text, in the realistic experimental setting the measurement data is unavoidably and significantly altered by SPAM errors. We can model those errors as invertible linear maps S and M, corresponding to state preparation and measurement errors, respectively, obtaining the noisy data model (see also Eq. (4))

$$y[l] = \frac{1}{2} M \exp(-\mathrm{i} t_l h) S. \tag{50}$$

This data model assumes that the initial and final ramp phases are to a good approximation particle number preserving. We allow for S, M to be general invertible linear maps rather than restrict them to being unitary in order to model incoherent effects during the ramping phases. In the presence of incoherent effects our model describes the behaviour of the dominant eigenvector of the density matrix.

In the following we outline the algorithmic strategies we use in order to alleviate the effect of S and M on the recovery. We begin in Section IV A by discussing the pre-processing step that removes *either* the state preparation *or* the measurement (SPOM) error from the data. In fact, this strategy enables us to fully characterize SPOM errors in the post-processing of the Hamiltonian identification. In Section IV B, we subsequently discuss in detail the remaining error which we take—without loss of generality—to be the measurement ramp.

A. Removing and characterizing SPOM errors

To discuss SPOM errors, we consider data with *either* state preparation *or* measurement errors, which has the form

$$y[l] = \frac{1}{2} M \exp(-\mathrm{i} t_l h), \text{ or} \tag{51}$$

$$y[l] = \frac{1}{2} \exp(-\mathrm{i} t_l h) S. \tag{52}$$

Without loss of generality, here we discuss state preparation errors, i.e., data of the form (52). Our conclusions follow analogously for SPOM errors described by a final map M.

To begin with, let us write the data (52) in eigendecomposition as

$$\begin{aligned} y[l] &= \frac{1}{2} \exp(-\mathrm{i} t_l h) S \\ &= \frac{1}{2} \sum_{k=1}^{N} \mathrm{e}^{-\mathrm{i} t_l \lambda_k} \left| v_k \right\rangle\!\left\langle v_k \right| S. \end{aligned} \tag{53}$$

Observe that, as in the error-free case, each coefficient matrix $\left| v_l \right\rangle\!\left\langle v_l \right| S$ has unit rank.

This allows us to remove the initial map from the data series in the pre-processing of the identification algorithm by forming the data series

$$y^{(l_0)}[l] = y[l](y[l_0])^{+} = \sum_{k=1}^{N} e^{-i\lambda_k (t_l - t_{l_0})} \left| v_k \right\rangle\!\left\langle v_k \right|. \tag{54}$$

Algorithm 5 $\mathrm{SPOMremoval}(y, s, w)$

Input: $y \in \mathbb{C}^{(L+1)\times N\times N}$, $s \le L$, $w \le L$.
for $l \in [\lfloor L/s \rfloor]$ **do**
  Calculate $P = (y[ls])^{+}$.
  for $k \in [ls - w, ls + w]$ **do**
    Calculate $y^{(l)}[k] = y[k] \cdot P$.
  end for
end for
Output: the concatenation $y_{\text{tot}} = (y^{(1)}, y^{(2)}, \ldots, y^{(\lfloor L/s \rfloor)})$.

In doing so, all entries of the data series are now affected by the noise corrupting $y[l_0]$. To improve noise robustness, we concatenate the data series for various l_0 to obtain, given integers $s, w \le L$, the extended data series

$$y_{\text{tot}} = (y^{(0)}, y^{(s)}, y^{(2s)}, \ldots, y^{(\lfloor L/s \rfloor s)}), \tag{55}$$

where each $y^{(l_0)}[l]$ is restricted to $l \in [l_0 - w, l_0 + w]$. We summarize the SPOM error removal algorithm in Algorithm 5.

The algorithm performs Ls^{-1} inversions of $N \times N$ matrices and $(2w+1)Ls^{-1}$ multiplications, requiring $\mathcal{O}(Ls^{-1}wN^3)$ flops in total. The resulting data series gets inflated to size $\mathcal{O}(s^{-1}wL)$. Choosing larger values of s and smaller values of w, thus, controls not only the time and storage complexity of the SPOM removal algorithm but also of the consecutive algorithmic steps of the identification algorithm. Executing algorithm 5 on $y[l]^T$ instead of $y[l]$ as an input and transposing the matrices in the returned time series removes errors in the measurement instead of the state preparation.

SPOM error characterization. Using the input data y_{tot}, in which the initial map has been removed, in the two-step Hamiltonian reconstruction algorithm, we obtain an estimate for the Hamiltonian $\hat{h}$. We can use $\hat{h}$ to obtain a tomographic estimate of the initial map S via

$$\hat{S} = \frac{2}{L+1} \sum_{l=0}^{L} \exp[\mathrm{i} t_l \hat{h}] y[l], \tag{56}$$

or alternatively of the final map M if we chose to remove it from y_{tot} instead of S.

B. The measurement error

Algorithm 5 removes either the initial or the final map from the data. Removing the initial map still leaves us with the final ramp M as a source of systematic error, the effect of which we discuss in the following. This error in fact explains the systematic error of the method observed in the experiments presented in the main text.

When the initial map is a general invertible matrix with no further restrictions, it is impossible to uniquely identify an arbitrary final map at the same time. This is because our model (50) contains a gauge freedom; a simultaneous transformation of S, M that leaves the data $y[l]$ invariant. As argued in the

paragraph *Imbalance between initial and final ramping phase* in Methods C, we expect the final ramp M to be nearly diagonal. This provides us with additional structure. Note that even if the diagonality assumption does not hold exactly, the gauge freedom allows us to partially enforce it.

The frequency estimation is robust against systematic errors due to the non-trivial final map. The systematic errors the identification therefore originate from the eigenspace reconstruction step. Deriving analytical expressions for how the measurement error biases the result of the non-convexly constrained optimization problem (28) and algorithm 4 is not straight-forward. For this reason, we instead focus on the relaxation to the linear inversion problem (29) with the unique solution $\Pi = \left(A^\lambda\right)^+ Y$. Let Y_0, Π_0 be the input data and solution to (29) in the absence of a final map. Including M, the data has the form $Y = Y_0(M^T \otimes M^{-1})$ and, thus, the solution becomes $\Pi = \Pi_0(M^T \otimes M^{-1})$. Note that here we have removed any initial ramp from the data using Algorithm 5. In Algorithm 3, we project the rows of Π to real-valued unit-rank projectors, to obtain the eigenspaces P_k of $\tilde{h}$. In the case of a diagonal unitary $M = \operatorname{diag}(e^{i\phi_1}, \dots, e^{i\phi_N})$, the rows of Π are already rank-one projectors and, taking the real part, the eigenspace estimates are $P_k = C(\phi) \circ |v_k\rangle\langle v_k|$ with $\circ$ the Hadamard product (entry-wise multiplication) and

$$C(\phi) = \begin{bmatrix} 1 & \cos(\phi_1 - \phi_2) & \dots & \cos(\phi_1 - \phi_N) \\ \cos(\phi_1 - \phi_2) & 1 & & \\ \vdots & & \ddots & \vdots \\ \cos(\phi_1 - \phi_N) & & \dots & 1 \end{bmatrix}. \tag{57}$$

Hence, also the recovered Hamiltonian becomes

$$\tilde{h} = C(\phi) \circ h_0 \,. \tag{58}$$

We find that the recovery is exact on the diagonal and there is a systematic error in the sign and amplitude of the off-diagonal terms. In Section VII A we estimate the magnitude of this error in the case where M is given by a linear model of the final ramping phase using analytical and numerical evidence. Furthermore, in Section VIII we propose an algorithm that removes the sign part of the systematic error under mild assumptions on the quality of the implementation of the target Hamiltonian.

V. THE COMPLETE IDENTIFICATION ALGORITHM

Algorithm 6 summarizes the complete algorithm for Hamiltonian reconstruction. The input to the algorithm are the data y, the support of the Hamiltonian model Ω and the time grid spacing Δt of the samples. The step size s and the window size w control the SPOM removal Algorithm 5. Both ESPRIT algorithms require a Hankel dimension K, tensorESPRIT further requires the sample set $\mathcal{S}$. The non-convex reconstruction using a conjugate gradient descent additionally requires a gradient tolerance ϵ, an initial point Q_0 and a regularization parameter μ. As demonstrated in the numerical benchmarks, Q_0 can be chosen at random. The success rate can be additionally improved by restarting the algorithm from another random initialization if the objective function is above a predefined threshold. For the recovery from experimental data, we initialize Q_0 at the eigenprojectors of the targeted Hamiltonian.

Algorithm 6 $\operatorname{HamRec}(y, \Delta t, s, w, K, [\mathcal{S}], [\epsilon, Q_0, \mu, \Omega])$

Input: Data $y \in \mathbb{C}^{(L+1)\times N\times N}$, sample rate $1/\Delta t$, SPOM removal parameters s, w, Hankel dimension K, [*for* tensorESPRIT: sample set $\mathcal{S}$], [*for* conjGrad: tolerance ϵ, initialization Q_0, regularization μ, support Ω]

1: $y_{\text{total}} = \operatorname{SPOMremoval}(y, s, w)$.
2: Extract the eigenfrequencies using $z = \operatorname{ESPRIT}(\operatorname{Tr} y_{\text{total}}, K, s, w)$, or using $z = \operatorname{tensorESPRIT}(y, K, \mathcal{S})$.
3: $\lambda_k = -\operatorname{Im}(\log(z_k))/(\Delta t)$ for $k \in [N]$.
4: Calculate matrix A^λ depending on s, w.
5: Reconstruct the eigenspace projectors either using $\Pi = \operatorname{linInvPP}(y_{\text{total}}, \mathcal{A}^\lambda)$ or $Q = \operatorname{conjGrad}(f_{y_{\text{total}}, A^\lambda, \mu}, Q_0, \epsilon)$ and set $\Pi = \Pi(Q)$.
6: Set $\hat{h} = H^\lambda(\Pi)$.
7: Set $\hat{S}$ according to Eq. (56).

Output: Hamiltonian coefficient matrix $\hat{h}$, initial map $\hat{S}$.

Algorithm 6 as stated here is robust against errors in the state-preparation and returns an estimate for $\hat{S}$. As explained in Section IV, we arrive at a variant of Algorithm 6 that is robust under errors in the measurement by applying the SPOM-removal algorithm to the transposed data and estimate $\hat{M}$ (instead of $\hat{S}$) in the last step.

A. Estimating the time complexity

Let us summarise the time complexity of the individual steps of the reconstruction algorithm: SPOM removal takes $\mathcal{O}(s^{-1}wLN^3 + L^2N)$ flops. The ESPRIT algorithm on the result of the SPOM-removal step as input requires $\mathcal{O}(s^{-1}wLN^2)$ flops, alternatively tensorESPRIT, which does not use the SPOM-removal step, requires $\mathcal{O}((LN^2 + |S|)N^3)$ flops. Post-projected linear inversion contributes $\mathcal{O}(s^{-1}wLN^2 + N^3)$ flops. Run-time estimates for the non-convex conjugate gradient algorithm are more involved as they depend on speed of convergence of the decent algorithm and of the matrix exponentiation. We suspect that the complexity scales linearly in $s^{-1}wL$ and as a low-degree polynomial in N. Thus, we expect that it is not dominating the parametric dependence of the run-time. Roughly speaking, the quadratic 'blow-up' of the data in the SPOM-removal step (assuming $w \in \mathcal{O}(L)$, $s \in \mathcal{O}(1)$) and choosing $|\mathcal{S}| \in \mathcal{O}(L^2)$ in tensorESPRIT causes all algorithmic steps to scale at most as $\mathcal{O}(L^2N^3)$. This step also determines the required storage to be in $\mathcal{O}(s^{-1}wLN^2)$.

B. Increasing the predictive power

When benchmarking the performance of the algorithms in numerical simulations, where the 'true' Hamiltonian generating the data is known a priori, it is instructive to consider not only the recovery error of the Hamiltonian itself but also the achieved fitting error to the data. Due to the gauge freedom in the SPAM data model (50) different tuples (h, S, M) give rise to the same observed data. For this reason, the fitting error is more suitable to draw conclusions about the capabilities of an estimate $(\hat{h}, \hat{S}, \hat{M})$ to predict further time series data with the same ramping phase—in a strict reading of terminology, shifting from the question of Hamiltonian identification to the question of Hamiltonian learning. We refer to

$$\mathcal{E}_{\text{pred}} := \frac{1}{N\sqrt{L+1}} \sum_{l=0}^{L} \sqrt{\|\hat{y}[l] - y[l]\|_{\ell_2}^2}, \qquad (59)$$

as the *prediction error* in the following.

We find that we can further reduce the prediction error the recovered model exhibits by running our recovery procedure iteratively multiple times, alternatingly optimizing the initial and the final map. After running Algorithm 6 once, we obtain the estimate $(\hat{h}^{(0)}, S^{(0)}, M^{(0)} = \mathrm{Id})$, update the data to

$$y^{(1)}[l] = (y[l](\hat{S}^{(0)})^{-1})^t \qquad (60)$$

and rerun the reconstruction algorithm. We, thus, obtain a new tuple $(\hat{h}^{(1)}, \hat{S}^{(1)} = \mathrm{Id}, \hat{M}^{(1)})$. Now updating the data series to

$$y^{(2)}[l] = (\hat{M}^{(1)})^{-1} y^{(1)}[l] \qquad (61)$$

and rerunning the algorithm yields a tuple $(\hat{h}^{(2)}, \hat{S}^{(2)}, \hat{M}^{(2)} = \mathrm{Id})$ and so forth. The final estimate $(\hat{h}, \hat{S}, \hat{M})$ of (h, S, M) can be computed from the tuples $(\hat{h}^{(i)}, \hat{S}^{(i)}, \hat{M}^{(i)})$ for $i \in [0, \ldots, r]$ via

$$\hat{h} = \hat{h}^{(r)}, \quad \hat{S} = \hat{S}^{(r)} \ldots \hat{S}^{(0)}, \quad \hat{M} = \hat{M}^{(0)} \ldots \hat{M}^{(r)}. \qquad (62)$$

We present numerical results on this iterative procedure in Section VII A. We find that already one iteration improves the systematic prediction error significantly, while having little effect on the systematic analog implementation error.

VI. NUMERICAL BENCHMARKS

We here conduct a detailed analysis of the performance of the various stages of our algorithm on simulated data. The Hamiltonian simulation and algorithm are implemented in the Python language. For frequency extraction, the Hankel dimension is set to $K = \lfloor L/2 \rfloor$. Note that this increases the computational complexity of the algorithms compared to the optimal choice of $K \in \mathrm{O}(N)$. For tensorESPRIT the set $\mathcal{S}$ is chosen according to (23). The non-convex optimization is initialized with Q_0 drawn at random from the Haar measure on $\mathrm{O}(N)$ for the numerical tests. The success rate is additionally improved by restarting the algorithm from another random initialization if the objective function is above a predefined threshold. If the SPOM removal pre-processing step is applied, we use parameters $s = 5$ and $w = L$.

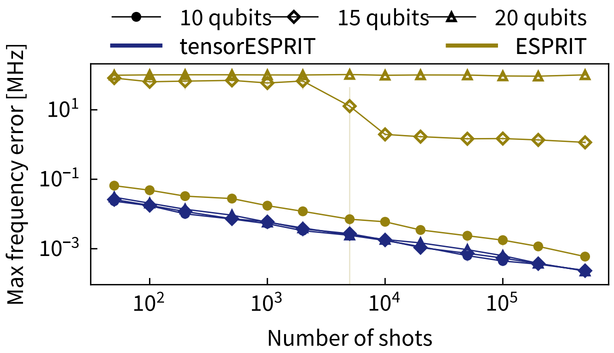

Supplementary Figure 1. **The ℓ_∞-error of the recovered spectrum of tensorESPRIT (blue) and ESPRIT (mustard) with varying levels of shot noise.** Every point is averaged over 10 random comb Hamiltonians with $N = 10$ (circles), 15 (diamonds) and 20 (triangles). The error bars represent the standard deviation and are mostly smaller than the markers. SPOM removal pre-processing (55) is applied. All markers of tensorESPRIT coincide.

A. Data models and Hamiltonian ensembles

We simulate the time evolution for total time $T = 0.6\,\mu\text{s}$ with sample rate $r = 1/\Delta t = 250\,\text{MHz}$ ($L = 150$) under the following ensembles of random non-interacting Hamiltonian:

- *Random comb Hamiltonians.* $h_{\text{comb}} = Q\,\mathrm{diag}(\lambda)Q^T$, where $\lambda = (\lambda_1, \ldots, \lambda_N)$ consists of equally spaced frequencies in the range $[-18.4, 17.0]\,\text{MHz}$ and Q is a Haar random orthogonal $N \times N$ matrix.
- *Random banded Hamiltonians.* Let ν be the uniform distribution on $[0, 20]\,\text{MHz}$. The diagonal entries $(h_{\text{banded}})_{k,k}$ are N independent samples from ν. The entries of the first off-diagonals $(h_{\text{banded}})_{k,k+1} = (h_{\text{banded}})_{k,k-1}$ are $N-1$ independent samples from ν. All other entries of h_{banded} are zero.
- *Random Harper Hamiltonians.* $(h_{\text{Harper}})_{k,k+1} = (h_{\text{Harper}})_{k,k-1} = -20\,\text{MHz}$ and $(h_{\text{Harper}})_{k,k} = 20\cos(2\pi k b)\,\text{MHz}$, where b is drawn uniformly at random from $[0, 1]$. All other $(h_{\text{Harper}})_{k,l}$ with $|k - l| > 1$ are zero.

Simulating noise and errors. We simulate the time series data according to (50) with initial and final ramps S and M computed using one of the following prescriptions:

- *Random unitary.* Drawn from the Haar measure on $U(N)$.
- *Random diagonal unitary.* $\mathrm{diag}(e^{i\phi_j})$, with $(\phi_j)_{j=1}^N$ i.i.d. samples from the uniform distribution over $[0, 2\pi)$.

System size N	5	20	50	100
ESPRIT [s]	2.0×10^{-2}	8.8×10^{-2}	1.1	9.9
tensorESPRIT [s]	5.3×10^{-2}	7.9×10^{-1}	9.0	81

Supplementary Table 1. **Run-times of ESPRIT and tensorESPRIT** on modern consumer grade SoC (Apple MacBook Air M1). Random comb Hamiltonians on various system sizes have been used to simulate $L = 150$ time steps. The run-times of ESPRIT include the SPOM removal pre-processing step (55) with algorithm parameters set to $s = 5, w = L$.

- *Constant-v model.* Ramp model from Section VII A. Idling frequencies of the qubits are drawn from the uniform distribution on $[-400, 100]$ MHz. For the benchmarks we set the parameters to $v = 790$ MHz/ns and $\tau = 0.05$ ns.

To account for shot noise induced by a finite number σ of samples for each expectation value, we replace each entry $y_{i,j}[l]$ by a sample from $\frac{1}{\sigma}(\mathrm{B}(\sigma, \mathrm{Re}\{y_{i,j}[l]\}) + \mathrm{i}\mathrm{B}(\sigma, \mathrm{Im}\{y_{i,j}[l]\}))$, where $\mathrm{B}(n,p)$ is the binomial distribution for n trials and probability of success p.

B. Frequency extraction

In this section, we benchmark the ability of ESPRIT and tensorESPRIT, introduced in Section II A and Section II B, to recover Hamiltonian frequencies from the simulated data. We demonstrate super-resolution capabilities of both algorithms. We find that tensorESPRIT is capable of recovering completely degenerate spectra with no decrease in accuracy, making it the more scalable approach.

We first examine the dependence of the recovery on the number of shots σ used to estimate each expectation value. We use both algorithms to recover the spectrum of random comb Hamiltonians with varying σ for three system sizes $N = 10$, 15, and 20. We set $S = M = \mathbb{1}$. The SPOM removal procedure (55) is still used to increase the signal-to-noise ratio. The l_∞-error of the recovered frequencies with respected to the their true values averaged over 10 Hamiltonian instances is plotted in Fig. 1. We find that the recovery error of tensorESPRIT scales as $\sigma^{-\frac{1}{2}}$. Using tensorESPRIT, all instances are recovered up-to shot noise limitation. For small system size $N = 10$ the recovery error of ESPRIT shows the same scaling and recovers all instances with comparable accuracy. For system sizes $N = 15$ and 20, ESPRIT has a large recovery error with a phase transition appearing for $N = 15$ at $\sigma = 5000$, above which some instances can be regarded as recovered. This can be explained by the fact that above $N = 15$ the frequency spacing of random comb Hamiltonian instances (with fixed bandwidth) is too narrow to be resolved by the ESPRIT algorithm.

To highlight this effect and demonstrate the stability of tensorESPRIT, we next examine the recovery of (nearly) degenerate spectra, with and without the effect of the SPOM removal (55). To this end we use random comb Hamiltonians

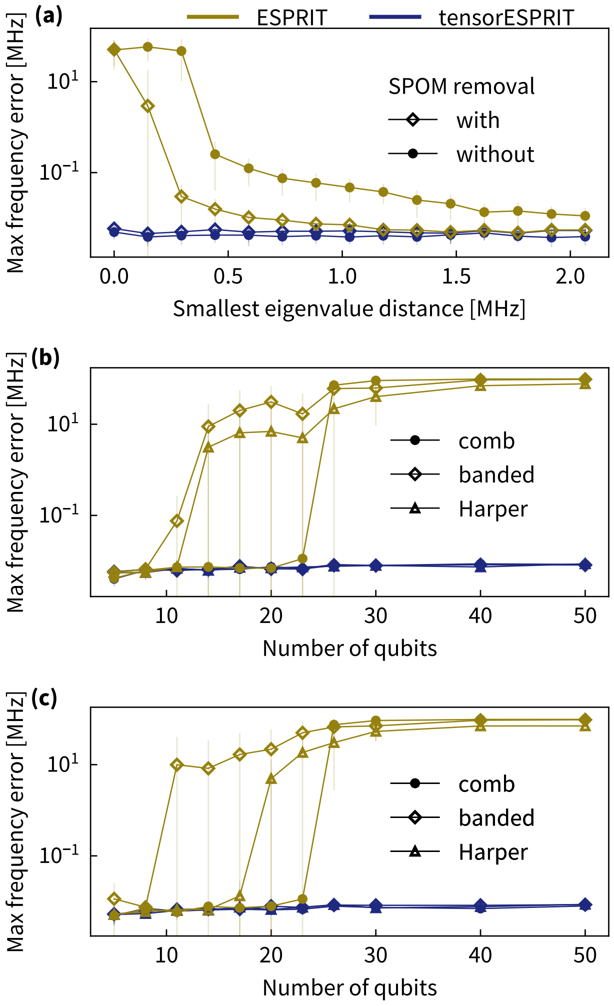

Supplementary Figure 2. **The ℓ_∞ recovery errors of tensorESPRIT (blue) and ESPRIT (mustard) for (nearly) degenerate spectra and different system sizes.** Shot noise with $\sigma = 1000$ is applied. The error bars represent the standard deviation. **(a)** Recovery of the spectra of random comb Hamiltonians ($N = 5$) with the second largest eigenvalue varied. Average over 30 runs with (diamonds) and without (circles) using SPOM removal pre-processing. x-axis displays the distance of the second and third largest eigenvalues. $S = M = \mathbb{1}$. **(b)** Average frequency recovery error over 10 runs of random comb, banded and Harper Hamiltonians versus the system size for $S = M = \mathbb{1}$ and **(c)** for Haar-random unitaries S and M. SPOM-removal step applied.

($N = 5$), where the second largest frequency is varied. Fig. 2, panel (a), displays the ℓ_∞ recovery error of both algorithms as a function of the distance between the second and the third largest frequencies. ESPRIT exhibits a phase transition and fails to recover spectra with small frequency spacing. Taking a look at the output spectra of the ESPRIT algorithm, we observe that ESPRIT misses one of the nearly degenerate frequencies and substitutes it with a frequency that originates in the noise subspace. Comparing the recovery with and without a preceding SPOM-removal step, we find that SPOM removal

System size N		5	10	25	50
linInvPP [s]	(a)	1.1×10^{-3}	4.3×10^{-3}	2.3×10^{-2}	1.4×10^{-1}
	(b)	2.2×10^{-3}	4.9×10^{-3}	2.2×10^{-2}	1.8×10^{-1}
conjGrad [s]	(a)	2.4×10^{-2}	8.4×10^{-2}	2.2	16.4
	(b)	8.7×10^{-2}	9.9×10^{-2}	1.6	2.3×10^{2}
regConjGrad [s]	(a)	4.5×10^{-2}	8.4×10^{-2}	1.8	40.0
	(b)	1.1×10^{-1}	1.6×10^{-1}	3.1	2.8×10^{2}

Supplementary Table 2. **Run-times of linear inversion with post-projection (linInvPP), conjugate gradient descent (conjGrad) and regularized conjugate gradient descent (regConjGrad)** on modern consumer grade SoC (Apple MacBook Air M1). Simulation with random banded Hamiltonians on varying system sizes with (a) no shot noise and (b) shot noise (σ = 1000). SPOM removal pre-processing (55) is applied.

significantly improves the resolution capabilities of ESPRIT, even though no SPOM error is present. In contrast, we see that neither the distance between the frequencies nor the SPOM removal step affect the recovery performance of tensorESPRIT. The effect of the SPOM-removal step on ESPRIT can be understood by the fact that this step makes all Fourier coefficients in $\mathrm{Tr}\, y_{\mathrm{tot}}$ equal to one (up to incoherent noise), which improves the performance of ESPRIT [9]. On the other hand it does not improve the performance of tensorESPRIT, since the pseudoinverse in forming the matrices $A^{(k,l,k',l')}$ already has similar impact.

The remaining panels of Fig. 2 show the performance in recovering the spectra of random comb, banded and Harper Hamiltonians of increasing system sizes, without (panel (b)) and with (panel (c)) SPOM errors. ESPRIT performs well only for small system sizes. The admissible system sizes depend on the Hamiltonian ensemble. The recovery of tensorESPRIT is successful also for large system sizes for all three Hamiltonian ensembles. We do not observe a deterioration of the recovery when including Haar random unitaries as SPOM matrices S and M for tensorESPRIT.

In summary, we conclude that ESPRIT is suitable for recovering spectra with sufficiently well-separated frequencies as is typically found for small system sizes. The shortcomings of ESPRIT in resolving degenerate spectra are resolved by tensorESPRIT, demonstrating consistent performance for larger system-sizes. This however comes at a cost of increased empirical computation times of tensorESPRIT compared to ESPRIT, Table 1.

C. Eigenspace reconstruction

In section Section III we proposed different methods to solve the optimization problem (24) in order to find the Hamiltonian eigenvectors, given eigenfrequencies recovered by ESPRIT or tensorESPRIT. We here compare the performance of linear inversion with post-projection (linInvPP), non-convex conjugate gradient descent over $\mathrm{O}(N)$ (conjGrad) and regularized conjugate gradient descent over $\mathrm{O}(N)$ (regConjGrad). The first two methods ignore the support constraint and solve

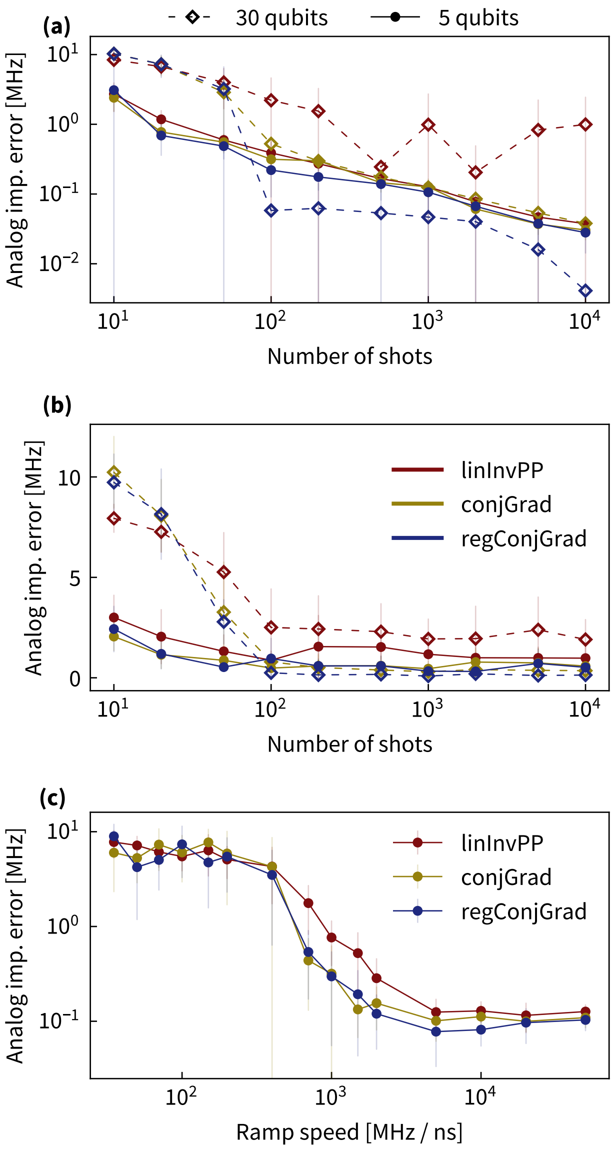

Supplementary Figure 3. **Eigenspace reconstruction of random Harper Hamiltonians** of size N = 5 (circles) and 30 (diamonds). Recovery error (analog implementation error metric) averaged over 10 instances for linear inversion with post-projection (linInvPP, red), conjugate gradient descent (conjGrad, mustard) and regularized conjugate gradient descent (regConjGrad, blue). Error bars indicate the standard deviation. Eigenfrequencies are extracted using tensorESPRIT, SPOM removal pre-processing (55) is applied. Recovery error for varying levels of shot noise **(a)** without SPAM errors ($S = M = \mathbb{1}$) and **(b)** for S a Haar-random unitary and M given by the constant-v model. **(c)** Effect of ramping speed in the constant-v model on the recovery (σ = 1000).

the remaining unconstrained problem. RegConjGrad imposes a relaxed support constraint via regularization. In this section, we benchmark these methods, in particular the effect of structure constraints on the robustness of the protocol against statistical and systematic errors.

First, we study how the number of shots σ used to estimate each expectation value impacts the performance of the eigenspace reconstruction methods. To this end, we simulate the time evolution under a random Harper Hamiltonian on 5 and 30 qubits and add varying levels of shot noise, before running the three recovery procedures. The distance between the recovered Hamiltonian and the one used in the simulation, the recovery error, is measured in terms of the analog implementation error (5) in the main text. The average recovery errors are displayed in Fig. 3 (a) without SPAM errors and (b) with SPAM errors included in the simulation. All reconstruction algorithms are able to recover the 5 qubit Hamiltonian. In the absence of SPAM errors, the recovery error is asymptotically compatible with a scaling as $\mathcal{O}(\sigma^{-\frac{1}{2}})$. For $N = 30$ regConjGrad has significantly better recovery results than the other methods. The error of conjGrad and regConjGrad exhibits a phase transition around $\sigma = 100$. A similar behaviour can be seen in the presence of SPAM errors. We, thus, conclude that for larger systems exploiting the support constraints improves the stability. At the same time linInvPP is most sensitive to the conditioning of the linear inverse problem for larger system sizes. We generally observe that the convergence becomes considerably more sensitive to the instances and initial condition when including the regularization term, hinting at a more rugged optimization landscape. Tuning the regularization parameter as described in Section III B can significantly improve the convergence here.

In Fig. 3 (c), we study the impact of SPAM errors originating from ramps with finite ramping speed on the recovery. See Section VII A for details on the ramping model used. We observe that only for ramp speed above roughly 1000 MHz/ns the recovery consistently succeeds for all algorithms while for slow rampings recovery generally fails. Importantly, both conjugate gradient methods are more robust than linInvPP. The experimentally observed speed in our setting is around 790 MHz/ns, which lies in the regime where the difference in the performance of the methods is most pronounced.

Next, we look at the impact of (near) spectral degeneracy of the Hamiltonian on the recovery in Fig. 4. To this end, we vary one eigenfrequency of a random comb Hamiltonian in panel (a). We find that for small frequency spacing, linInvPP has a comparatively large recovery error. In contrast, both non-convex optimization methods display consistently good recovery performance also for (near) degenerate spectra. For the recovery of Harper Hamiltonians on systems larger than 20 the performance of linInvPP decreases with the system size, panel (b), as here the spectrum becomes increasingly degenerate. Further, we see that the regularization reduces the systematic error, especially in large systems.

Table 2 details empirical run-times of the three algorithms for different system sizes and simulations with and without shot noise.

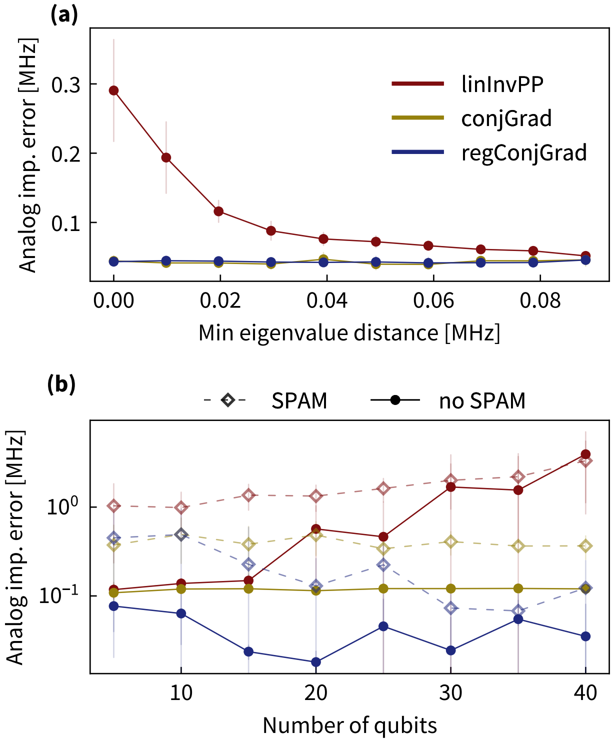

Supplementary Figure 4. **Recovery error of Hamiltonians with (nearly) degenerate spectra** for linInvPP (red), conjGrad (mustard), regConjGrad (blue) averaged over 10 instances. Error bars indicate standard deviation. We use tensorESPRIT for frequency extraction and apply SPOM removal pre-processing (55). **(a)** Data simulated with random comb Hamiltonians, $N = 10$, with the sixth smallest eigenvalue shifted towards the fifth smallest eigenvalue, plotting the analog implementation error against their distance. **(b)** Data simulated with random Harper Hamiltonians on various system sizes without SPAM errors (circles) and with Haar random unitary S and M given by the constant-v model (diamonds).

D. Compressed sensing capabilities

The linear inverse problem we solve for reconstructing the eigenvectors is typically highly over-determined. We measure $2(L+1)N^2$ real expectation values comprising y in order to infer the N^3 real parameters of Π. Taking time trace data, e.g., at sample rate 250 MHz for .6 μs the number of measurement times $2(L+1) = 2 \cdot 250 \cdot .6 = 300$ is considerably larger than the system size on current hardware with tens of sites.

Since the reconstruction algorithm additionally explicitly exploits restricted structure of the underlying signal, we expect that recovery is still possible with considerably less expectation values—following the paradigm of compressed sensing [20], even in the regime where the linear inversion problem becomes underdetermined.

We here numerically test the compressed sensing capabilities of the different algorithms. To this end we randomly subselect entries of $y[l]$ with a probability p for the eigenvector reconstruction. In expectation the number of (complex) measurement settings is, thus, reduced to $2(L+1)N^2p$. Fig. 5

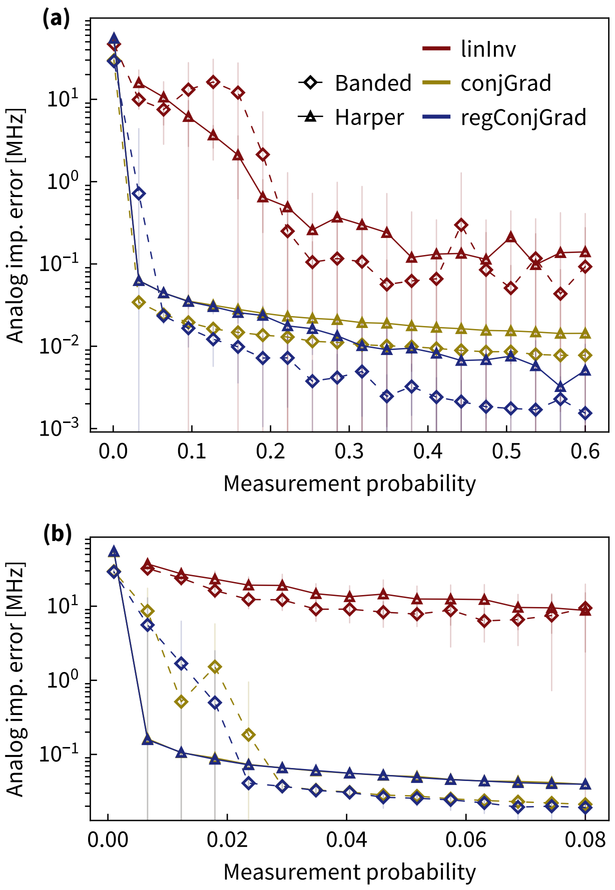

Supplementary Figure 5. **Compressed sensing recovery.** Recovery error averaged over 30 instances drawn from different Hamiltonian ensembles ($N = 20$) and for different eigenspace reconstruction algorithms, when measurement data is randomly subsampled. Error bars display the standard deviation. **(a)** a wider range of measurement probabilities; **(b)** in the regime of an under determined inverse problem. Simulation with $\sigma = 10^6$ shots and without SPAM errors.

displays the recovery error for different values of p. In particular, in the regime shown in panel (b), below $p = .07$ the problem becomes underdetermined as a linear inverse problem. We find that conjugate gradient algorithms can successfully recover Hamiltonian instances even when the problem (without structure assumptions) is underdetermined. The post-projected linear inversion algorithm in contrast does only allow for moderate subsampling of the measurement entries.

VII. ESTIMATING EXPERIMENTAL ERRORS

In this section, we explain how we empirically estimate the error on the Hamiltonian $\hat{h}$ and initial map $\hat{S}$ identified via the robust identification method Algorithm 6, including pre- and post-processing. This error comprises two contributions. First, it has a systematic contribution, which is due to the non-trivial final map $M \neq \mathbb{1}$ (Section VII A). Second, it has a statistical contribution due to the estimation of the expectation values Eq. (50) from finite statistics (Section VII B).

As noted in the main text, the impact of the systematic error on the predictive power of the identified Hamiltonian is reduced by the gauge freedom in (50) under simultaneous transformation of h, S, M. Additionally, the systematic error in the prediction error can be further reduced using the iterative procedure described in Section V B.

A. Systematic error: Final ramp effect estimation

To estimate the magnitude of the systematic error that is induced by a non-trivial final pulse ramping M, we use an idealized model of the final ramping phase with a constant ramping speed v and a padding time τ (*constant-v model*). In the experiment, ramping proceeds in three steps. First, we ramp the coupler frequencies to turn off the hopping term in the Hamiltonian. Second, to stabilize the frequencies, we let the system evolve under the (now diagonal) Hamiltonian for padding time τ, before finally ramping the qubits to their idling frequencies to enable their measurement. Our model assumes linear ramps with slope v of all the Hamiltonian entries. Hence, the final ramp is given by $M = U_{\text{coupler}} U_{\text{padding}} U_{\text{diag}}$, where

$$U_j := \mathcal{T} \exp\{-i \int_0^{\tau_j} H_j(t) dt\} \tag{63}$$

for $j \in \{\text{coupler}, \text{diag}\}$, with $\mathcal{T}$ denoting the time-ordering operator, and $U_{\text{padding}} = e^{-i\tau \operatorname{diag}(h)}$, where diag applied to a matrix returns its restriction to the diagonal. We set $H_{\text{coupler}}(t) = T_{\operatorname{diag}(h)}(h + \operatorname{sign}(\operatorname{diag}(h) - h) v t)$ and $H_{\text{diag}}(t) = T_{h_m}(\operatorname{diag}(h) + \operatorname{sign}(h_m - \operatorname{diag}(h)) v t)$. Here, h_m corresponds to the idle Hamiltonian at the end of the ramp pulse and the thresholding operator acts entry-wise as

$$T_g(x)_{i,j} = \begin{cases} \min\{(g)_{i,j}, x_{i,j}\} & \text{if } \operatorname{sign}(h_m - h)_{i,j} > 0, \\ \max\{(g)_{i,j}, x_{i,j}\} & \text{if } \operatorname{sign}(h_m - h)_{i,j} < 0. \end{cases} \tag{64}$$

The thresholding ensures that the entries of $H(t)$ stay equal to those of $\operatorname{diag}(h)$ and h_m respectively once they reach their final value in each ramping phase. The integration limits $\tau_{\text{coupler}}, \tau_{\text{diag}}$ are the minimal times at which all entries of $H_{\text{coupler}}(t), H_{\text{diag}}(t)$ reach $\operatorname{diag}(h), h_m$ respectively. We assume that the matrix after the ramp pulse h_m is a diagonal matrix with frequencies corresponding to the idling frequencies of the qubits.

Below, we empirically build trust in this model and estimate the model parameters. Using the empirically inferred model parameters, we estimate the systematic errors in the following way: Using the empirical estimates $\hat{h}, \hat{S}$ (the output of Algorithm 6), we simulate the time evolution using the model (50) with M given by the constant-v model. Running the identification Algorithm 6 again on the simulated data yields a second (bootstrapped) estimate $\tilde{h}, \tilde{S}$. We use $\mathcal{E}_{\text{analog}}(\hat{h}, \tilde{h})$ as an estimate for the systematic error of the analog implementation error. By comparing $\tilde{h}$ with $\hat{h}$ entrywise, we arrive at an entrywise systematic error estimate. Similarly, we can compute the systematic error in $\mathcal{E}_{\text{analog}}(\hat{S}, \mathbb{1})$ via $\mathcal{E}_{\text{analog}}(\hat{S}, \tilde{S})$.

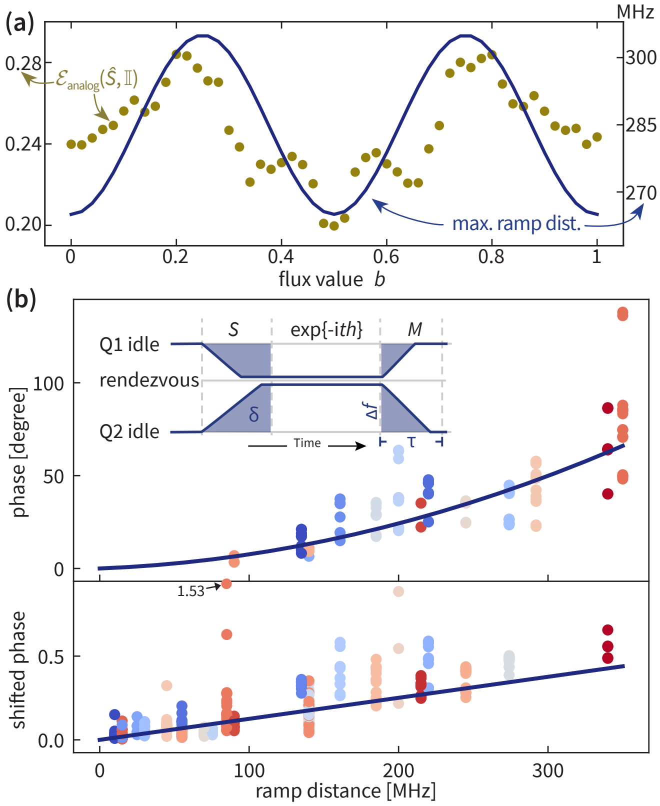

Supplementary Figure 6. **Validating the ramp model.** **(a)** Distance of the identified initial map before post-processing $\hat{S}'$ from the identity for the 5-qubit butterfly data of Fig. 6 of the main text (golden dots) and maximum ramp distance $\max_i |(h_0 - h_m)_{i,i}|$ (solid line) for each flux value $b \in [0,1]$. *Inset illustration of the ramp model.* The qubits initially at frequencies Q1 and Q2 are ramped to the common rendezvous frequency of 6500 MHz giving rise to an initial map S, where they evolve under the Hamiltonian h for time t until they are ramped back to their idle frequencies, giving rise to a final map M. The shaded areas show the total acquired phase δ during the ramp phases. **(b)** Phases accumulated on various connected 5-qubit subsets of the chip. *Top.* Phase accumulated on the qubit with maximum ramp distance from each subset. The fit is a quadratic function with zero offset, which gives estimates $v = 800 \pm 80$ MHz/ns, $\tau_{\text{tot}} = 0.09 \pm 0.03$ ns. *Bottom.* For the remaining qubits from each subset, shifted phase ξ given by (65) is plotted. The fit is a linear function with zero offset, which gives the estimate $v = 797 \pm 4$ MHz/ns.

Empirical validation of ramp model and parameter estimation. Our model for estimating the systematic error induced by the final ramping phase implies that the deviation of the initial and final ramp from the identity transformation depends on the ramp distance, that is, the absolute value of the entries of $h - h_m$. Indeed, the maximal ramp distance is expected to set the time-scale of the ramp phase and, thus, determines magnitude of the ramping effect in the data. In Fig. 6(a) we validate that, indeed, the deviation of the identified initial map $\hat{S}$ is proportional to the ramp distance $\max_{i,j} |(h - h_m)_{i,j}|$.

In order to give an estimate of the model parameters v and τ, we implement the zero Hamiltonian and reconstruct it with our identification method. In the rotating frame of the idle frequencies of the qubits, we effectively observe a diagonal Hamiltonian with eigenfrequencies that are the difference between the common rendezvous frequency (6500 MHz) and the idle frequencies. Since no couplers are involved, both the corresponding final and initial ramping maps are diagonal and contribute a complex phase to the data which is proportional to the combined surface area underneath the ramps, see the inset of Fig. 6(b).

Since the Hamiltonian is itself diagonal, it commutes with the final diagonal unitary so that, effectively, the data can be described as $y_{\text{diagonal}}(t) = \exp(-ith) MS$ with only SPOM error with an effective initial map MS present. We can thus determine the combined phase accumulated on each qubit during the true initial and final ramping directly as the phases of the diagonal entries of our estimate $\hat{S}$ of the effective initial map. Using the constant-v model for S and M, the magnitude of the total accumulated phase on the j-th qubit can be computed to be $|\phi_j| = 2\pi\Delta_j\,(\Delta_{\max}/v + \tau_{\text{tot}})$, where τ_{tot} is the sum of the padding times of the initial and final ramping phases and Δ_j is the ramping distance of the j-th qubit. $\Delta_{\max}$ is the maximum ramping distance in the set of qubits involved in the experiment and it sets the ramping time for all other qubits. In our experiment, we implemented the zero Hamiltonian on various 5-qubit connected subsets of the chip and hence $\Delta_{\max}$ differs for each subset.

To estimate the model parameters, in panel (b) of Fig. 6 we plot the magnitude of the phase accumulated on the qubit with the maximum ramping distance in the given subset against the maximum ramping distance for that subset. Fitting a quadratic function with zero offset gives us estimates $v_1 = 800 \pm 80$ MHz/ns and $\tau_{\text{tot}} = 0.09 \pm 0.03$ ns. Now we turn to the remaining qubits. Using the estimated padding time, we compute

$$\xi_j := \frac{\frac{|\phi_j|}{2\pi} - \tau_{\text{tot}}}{\Delta_{\max}} = \frac{1}{v}\Delta_j. \tag{65}$$

Using a linear fit we get a second ramp speed estimate $v_2 = 797 \pm 4$ MHz/ns, which is consistent with v_1 within experimental error, building trust in the model. Note that $v = 790$ MHz/ns and $\tau = 0.05$ ns (which is the part of τ_{tot} we assign to the final ramp) is the parameter value used in the numerical benchmarks of Section VI. To get a conservative estimate of the systematic errors, and in face of the variance in the data Fig. 6, we use $v = 350$ MHz/ns and $\tau = 0.1$ ns. The fit is shown in panels (b) and (c) of Fig. 6, where we excluded one qubit, which accumulated seemingly random phase in each run of the experiment.

Comparison to systematic error in numerical simulation. In order to build trust in our estimation method for the systematic error, we simulate the time evolution of random Harper Hamiltonians on varying system sizes with SPAM errors, and study how well the identification procedure performs in terms of analog implementation error of the recovered Hamiltonian and prediction error. We use random unitary initial maps and final maps that are either a random diagonal unitary or given by the constant-v model. Since the model with random diagonal unitaries can even change the sign of the recovered inter-

action strength, in this setting we make use of the additional post-processing step described in Section VIII. In Fig. 7, panels (a), (b) and (c), respectively, we plot the prediction error, the analog implementation error and the improvement of the errors when two iterations of the method are used, in the way described in Section V B.

When comparing these results to the experimental data Figs. 2, 5 and 6 in the main text, we see that the prediction errors are close to the prediction errors achieved by the constant-v model. This constitutes an independent validation of our method of estimating the systematic error in the Hamiltonian identification. In panel (c), we can see that the iterative procedure significantly improves the systematic error in the prediction error, while having little effect on the systematic error on the analog implementation error.

Scaling estimate of the systematic error. In panel (b) we observe that the systematic error decreases with the system size for the random diagonal unitary model for M. Even though not apparent in the parameter regime of the numerical benchmarking, we expect a similar dependence also for the constant-v model. This can be understood using the following theoretical reasoning. We have seen in Section IV B that in the absence of statistical errors and assuming a diagonal unitary $M = \mathrm{diag}(e^{i\phi_1}, \dots, e^{i\phi_N})$, the recovered Hamiltonian is given by (58). Suppose the case of a one-banded Hamiltonian with couplings of typical magnitude $\bar{J}$, where the diagonal entries have typical magnitude $\bar{h}$ and need to be ramped by a typical distance $\bar{\Delta}$. If we assume the linear ramping model above with constant ramping speed v and a padding time τ, we can estimate the magnitude of the systematic error in the analog implementation error[2] to be

$$\mathcal{E}_{\text{syst}} \approx \bar{J}(2/N)^{\frac{1}{2}} \left[1 - \cos\left(\pi \left(\frac{\Delta^2}{v} + 2\tau\bar{h} \right) \right) \right], \tag{66}$$

which using realistic values $\Delta = 300\,\text{MHz}$, $\bar{h} = \bar{J} = 20\,\text{MHz}$, $v = 350\,\text{HMz/ns}$, $\tau = 0.1\,\text{ns}$ evaluates to approximately $6.4\sqrt{2}/\sqrt{N}$ MHz, showing that the systematic error should decrease with system size for one-banded Hamiltonians, in accordance to Fig. 7. Note that this argument itself relies on relaxations of the original problem, assumptions on the final map M and the experimental characterization of the model. On this basis it further serves as a consistency check for the magnitude of the reported systematic errors.

Remaining sign-errors. As reported in the main text, we observe that the Hamiltonian identification algorithm recovers some interactions with the opposite sign than the target Hamiltonian. Such an error can be explained by the presence of a diagonal unitary final map with phase differences $\pi/2$ and $3\pi/2$. However, we find that the constant-v ramp model with the empirically estimated parameters and in the regime of the observed prediction error does never produce final maps with such large phase differences. Furthermore, the random diagonal ramp model that produces matrices M with sufficient phase differences to flip signs in the recovered interactions yields prediction errors that are considerably larger than the ones we observe in the experiment, see Fig. 7. The observed sign flips, thus, point to a separate source of sign systematic error. Under the assumption that sign flips originate from SPAM, we can however efficiently correct for them in the post-processing. We explain the corresponding post-processing algorithm for reconstructing M restricted to an orthogonal diagonal rotation in Section VIII.

[2] Here we are assuming that all the qubits are ramped in the same direction, which is close to reality, and neglect the ramping of the couplers.

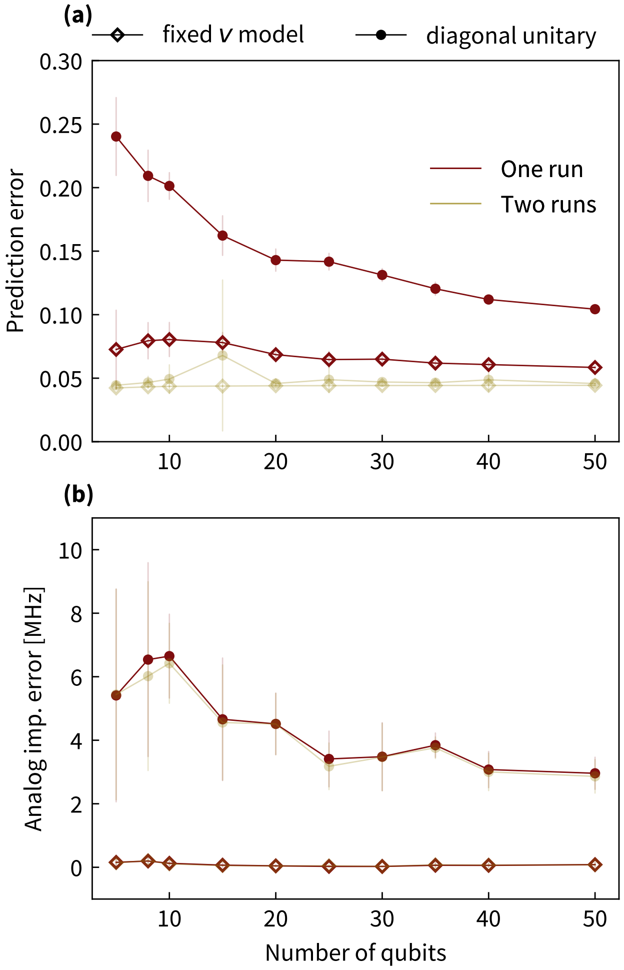

Supplementary Figure 7. **Systematic recovery and prediction error due to non-trivial final map.** Recovery of random Harper Hamiltonians on various system sizes, using one (red) and two (mustard) runs of the algorithm (see Section V B for details). SPAM errors modeled by a random unitary S and either a random diagonal unitary M (circles) or M given by the constant-v model (diamonds). The shot noise corresponds to $\sigma = 1000$ shots per expectation value. TensorESPRIT and regularized conjugate gradient descent are used in the recovery. The error bars represent the standard deviation over 10 instances. **(a)** Prediction error (59) of the recovered model. **(b)** Analog implementation error of the recovered Hamiltonian.

Algorithm 7 OneBandedCorrectFlips(h, h_0)

Input: symmetric $h \in \mathbb{R}^{N\times N}$, symmetric one-banded $h_0 \in \mathbb{R}^{N\times N}$.

```
Set D_M = 𝟙.
Define G_±(A) = A h A^T ± h_0.
for m ∈ {2, ..., N} do
    Set a_0 = |G_-(D_M)[m-1, m]|.
    Set a_flip = |G_+(D_M)[m-1, m]|.
    if a_flip < a_0 then
        Set (D_M)[m, m] = -1.
    end if
end for
Set ĥ = D_M h D_M.
```

Output: sign-fixed Hamiltonian coefficient matrix $\hat{h}$, final map estimate D_M.

B. Statistical error: Bootstrapping

Let us now turn to estimating the statistical error of the identification result. We estimate the size of the error induced on the Hamiltonian estimate that is returned by the identification method via parametric bootstrapping. To this end, we simulate time series data with finite statistical noise according to the model (50) with $M = \mathbb{1}$ using the identified Hamiltonian $\hat{h}$ and a Haar-random unitary for the initial ramp S. We then run the Hamiltonian identification method with conjGrad on 10^5 instances of such synthetic data. As the statistical error of the entry we use the 0.99-quantile (99% confidence level) of the absolute deviation of each entry in the Hamiltonians obtained from the synthetic data, from the corresponding entry of the identified Hamiltonian used to generate the data. We observe that the statistical errors of the entries are of comparable size and only report the maximal statistical error over all entries.

We also calculate 0.99-quantile of the deviation of the synthetically identified Hamiltonian $\hat{h}_{\text{bt}}$ from the originally identified Hamiltionian $\hat{h}$ in terms of the analog implementation error $\mathcal{E}_{\text{analog}}(\hat{h}_{\text{bt}}, \hat{h})$, (see Eq. (5) of the main text), and likewise for the eigenfrequencies. This is used as the statistical error estimate for the analog implementation error benchmark.

Omitting the regularization in the identification method reduces the computational complexity of the bootstrapping and produces more well-behaved empirical distributions of the deviation error. At the same time the regularization is shown in Section VI C to improve the estimate and, thus, the statistical error obtained in this way is expected to dominate the statistical error of the regularized identification method.

VIII. RECONSTRUCTING DIAGONAL ORTHOGONAL FINAL MAPS

We partially remove the systematic error induced by the final ramping phase with the following post-processing procedure. Suppose we are given the estimates $\hat{h}, \hat{S}$ by the identification Algorithm 6. To remove the sign part of the systematic

Algorithm 8 GreedyCorrectFlips(h, h_0)

Input: symmetric $h \in \mathbb{R}^{N\times N}$, symmetric $h_0 \in \mathbb{R}^{N\times N}$.

```
for m ≠ n in the order of decreasing |h[m, n]|, s.t. neither m nor
  n has been probed previously do
    Set a_0 = |G_-(D_M)[m, n]|.
    Set a_flip = |G_+(D_M)[m, n]|.
    if a_0 < a_flipped then
        Set D_M^flip = D_M.
        Set D_M^flip[m, m] = D_M^flip[n, n] = -1.
        if ||G_-(D_M^flip)||_F < ||G_-(D_M)||_F then
            Set D_M[m, m] = D_M[n, n] = -1.
        end if
    else
        Set D_M^1 = D_M^2 = D_M.
        Set D_M^1[m, m] = -1, D_M^2[n, n] = -1.
        if ||G_-(D_M^1)||_F < ||G_-(D_M^2)|| then
            Set D_M[m, m] = -1.
        else
            Set D_M[n, n] = -1.
        end if
    end if
end for
Set ĥ = D_M h D_M.
```

Output: sign-fixed Hamiltonian coefficient matrix $\hat{h}$, final map estimate D_M.

error on the Hamiltonian recovery, we determine an orthogonal diagonal $\hat{M} = \hat{D}_M$ that solves

$$\underset{D_M = \text{diag}\,\pm 1}{\text{minimize}} \quad \|D_M \hat{h} D_M - h_0\|_F^2 \tag{67}$$

and perform the gauge transformation

$$\begin{aligned} \hat{h}' &= \hat{D}_M \hat{h} \hat{D}_M, \\ \hat{S}' &= \hat{D}_M \hat{S}, \end{aligned} \tag{68}$$

to obtain the model $(\hat{h}', \hat{S}', \hat{D}_M)$. This estimate further reduces the systematic errors compared to $(\tilde{h}, \tilde{S}, \mathbb{1})$.

Note that in general solving (67) is an NP-hard problem. To see this, consider the case when h_0 is a matrix with all ones and $\hat{h}$ is a matrix with entries ± 1. The problem then encodes the maximum balanced subgraph problem, which is known to be NP-hard [21]. However, for 1D nearest-neighbour hopping Hamiltonians, where the Hamiltonian coefficient matrices are one-banded, we can give an efficient algorithm: The algorithm sets $\hat{D}_M[1,1] = +1$ and then updates the diagonal entries of $\hat{D}_M$ one-by-one. For the element $\hat{D}_M[m,m]$ with $m > 1$, it decides to set it to ± 1, picking in each turn the option more favourable to the cost function. Since in each of the $N-1$ steps we take into account one more independent off-diagonal element of h_0, of which there are also exactly $N-1$, this procedure finds the exact solution in the one-banded case. The algorithm is summarized in Algorithm 7.

For general Hamiltonian, one can apply the following greedy heuristic algorithm: We create a list of off-diagonal elements of $\tilde{h}$, ordered according to their decreasing absolute

value. Then, we set $\hat{D}_M = \mathbb{1}$ and loop over the entries of the list. At each step, the algorithm decides whether flipping the sign of the element $h[m,n]$ under consideration would decrease the cost function. If yes, it sets either $\hat{D}_M[m,m] = -1$ or $\hat{D}_M[n,n] = -1$, depending on which one is better with respect to the cost function. If not, it either does nothing or sets both $\hat{D}_M[m,m] = \hat{D}_M[n,n] = -1$, depending on which option is better with respect to the cost function. The algorithm is summarized in Algorithm 8. We note that for the problem sizes we encounter in the experiment we can also exactly solve the minimization problem (67) through exhaustive search.

---

[1] C. E. Shannon, *Communication in the Presence of Noise*, Proceedings of the IRE **37**, 10 (1949).

[2] P. R. Prony, *Essai experimentale et analytique*, J. de l'Ecole Polytechnique **1**, 24 (1795).

[3] R. Schmidt, *Multiple emitter location and signal parameter estimation*, IEEE Trans. Ant. Prop. **34**, 276 (1986).

[4] R. Roy and T. Kailath, *ESPRIT-estimation of signal parameters via rotational invariance techniques*, IEEE Transactions on Acoustics, Speech, and Signal Processing **37**, 984 (1989).

[5] E. J. Candès and C. Fernandez-Granda, *Super-resolution from noisy data*, J. Fourier An. App. **19**, 1229 (2013).

[6] E. J. Candes and C. Fernandez-Granda, *Towards a mathematical theory of super-resolution*, Comm. Pure App. Math. **67**, 906 (2014).

[7] R. Roy, A. Paulraj, and T. Kailath, Estimation of signal parameters via rotational invariance techniques-ESPRIT, in *MILCOM 1986-IEEE Military Communications Conference: Communications-Computers: Teamed for the 90's*, Vol. 3 (IEEE, 1986) pp. 41–6.

[8] A. Fannjiang, *Compressive spectral estimation with single-snapshot Esprit: Stability and resolution*, arXiv:1607.01827.

[9] W. Li, W. Liao, and A. Fannjiang, *Super-resolution limit of the ESPRIT algorithm*, IEEE Trans. Inf. Th. **66**, 4593 (2020), arXiv:1905.03782.

[10] G. H. Golub and C. F. van Loan, *Matrix computations* (The Johns Hopkins University Press, Baltimore, 1989).

[11] J. C. Bridgeman and C. T. Chubb, *Hand-waving and interpretive dance: An introductory course on tensor networks*, J. Phys. A **50**, 223001 (2017), arXiv:1603.03039.

[12] N. Halko, P.-G. Martinsson, and J. A. Tropp, *Finding structure with randomness: Probabilistic algorithms for constructing approximate matrix decompositions*, (2010), arxiv:0909.4061.

[13] A. Edelman, T. A. Arias, and S. T. Smith, *The geometry of algorithms with orthogonality constraints*, SIAM J. Matr. Ana. App. **20**, 303 (1998).

[14] P.-A. Absil, R. Mahony, and R. Sepulchre, *Optimization algorithms on matrix manifolds* (Princeton University Press, 2009) google-Books-ID: NSQGQeLN3NcC.

[15] I. A. Luchnikov, A. Ryzhov, S. N. Filippov, and H. Ouerdane, *QGOpt: Riemannian optimization for quantum technologies*, SciPost Phys. **10**, 079 (2021).

[16] I. Luchnikov, M. Krechetov, and S. Filippov, *Riemannian geometry and automatic differentiation for optimization problems of quantum physics and quantum technologies*, New J. Phys. **23**, 073006 (2021).

[17] D. Hangleiter, I. Roth, D. Nagaj, and J. Eisert, *Easing the Monte Carlo sign problem*, Science Adv. **6**, eabb8341 (2020).

[18] C. Krumnow, L. Veis, Ö. Legeza, and J. Eisert, *Fermionic orbital optimization in tensor network states*, Phys. Rev. Lett. **117**, 210402 (2016).

[19] T. Abrudan, J. Eriksson, and V. Koivunen, *Conjugate gradient algorithm for optimization under unitary matrix constraint*, Signal Processing **89**, 1704 (2009).

[20] E. Candes and M. Wakin, *An introduction To compressive sampling*, IEEE Signal Process. Mag. **25**, 21 (2008).

[21] J. J. Bartholdi, *A good submatrix is hard to find*, Operations Research Letters **1**, 190 (1982).
